# The Inactivation of *Arx* in Pancreatic α-Cells Triggers Their Neogenesis and Conversion into Functional β-Like Cells

**DOI:** 10.1371/journal.pgen.1003934

**Published:** 2013-10-31

**Authors:** Monica Courtney, Elisabet Gjernes, Noémie Druelle, Christophe Ravaud, Andhira Vieira, Nouha Ben-Othman, Anja Pfeifer, Fabio Avolio, Gunter Leuckx, Sandra Lacas-Gervais, Fanny Burel-Vandenbos, Damien Ambrosetti, Jacob Hecksher-Sorensen, Philippe Ravassard, Harry Heimberg, Ahmed Mansouri, Patrick Collombat

**Affiliations:** 1 Université de Nice Sophia Antipolis, iBV, UMR 7277, Nice, France; 2 Inserm, iBV, U1091, Nice, France; 3 CNRS, iBV, UMR 7277, Nice, France; 4 Diabetes Research Center, Vrije Universiteit Brussel, Brussel, Belgium; 5 Centre Commun de Microscopie, Université de Nice Sophia Antipolis, Nice, France; 6 Laboratoire central d'Anatomie Pathologique, CHU de Nice, Nice, France; 7 Hagedorn Research Institute, Department of Developmental Biology, Gentofte, Denmark; 8 Biotechnology and Biotherapy Laboratory, Centre de Recherche de l'Institut du Cerveau et de la Moelle, CNRS UMR 7225; INSERM UMRS 975; University Pierre et Marie Curie, Hôpital Pitié Salpétrière, Paris, France; 9 Max-Planck Institute for Biophysical Chemistry, Department of Molecular Cell Biology, Göttingen, Germany; 10 Department of Clinical Neurophysiology, University of Göttingen, Göttingen, Germany; 11 Genome and Stem Cell Center, GENKOK, Erciyes University, Kayseri, Turkey; Harvard Medical School, United States of America

## Abstract

Recently, it was demonstrated that pancreatic new-born glucagon-producing cells can regenerate and convert into insulin-producing β-like cells through the ectopic expression of a single gene, *Pax4*. Here, combining conditional loss-of-function and lineage tracing approaches, we show that the selective inhibition of the *Arx* gene in α-cells is sufficient to promote the conversion of adult α-cells into β-like cells at any age. Interestingly, this conversion induces the continuous mobilization of duct-lining precursor cells to adopt an endocrine cell fate, the glucagon^+^ cells thereby generated being subsequently converted into β-like cells upon *Arx* inhibition. Of interest, through the generation and analysis of *Arx* and *Pax4* conditional double-mutants, we provide evidence that *Pax4* is dispensable for these regeneration processes, indicating that Arx represents the main trigger of α-cell-mediated β-like cell neogenesis. Importantly, the loss of *Arx* in α-cells is sufficient to regenerate a functional β-cell mass and thereby reverse diabetes following toxin-induced β-cell depletion. Our data therefore suggest that strategies aiming at inhibiting the expression of *Arx*, or its molecular targets/co-factors, may pave new avenues for the treatment of diabetes.

## Author Summary

Type 1 diabetes is a condition that results from the loss of insulin-producing β-cells. Despite current therapies, diabetic patients are prone to vascular complications. Using the mouse as a model, we previously found that pancreatic glucagon-expressing cells can be regenerated and converted into β-like cells by the forced expression of a single gene, *Pax4*. Here, we generated transgenic mice allowing both the permanent labeling of α-cells and the inactivation of *Arx* solely in this cell subtype. Our results indicate that, upon *Arx* inactivation, α-cells can be continuously regenerated from duct-lining precursors and converted into β-like cells. Importantly, the additional loss of *Pax4* does not impact these processes, suggesting that *Arx* is the main trigger of α-cell-mediated β-like cell neogenesis. Most interestingly, upon chemical induction of diabetes/β-cell loss, while control animals die or remain severely hyperglycemic, a normalization of the glycemia, a clear regeneration of the β-like cell mass, and an extended lifespan are noted in animals with the conditional inactivation of *Arx*. Our data therefore suggest that strategies aiming at inhibiting the expression of *Arx*, or its molecular targets/co-factors, may pave new avenues for the treatment of diabetes.

## Introduction

The mature pancreas is organized into two compartments with distinct functions: the exocrine pancreas is involved in the production of digestive enzymes and their transport into the duodenum via a branched ductal tree, whereas the endocrine compartment consists of highly vascularized functional units called islets of Langerhans. These contain five distinct cell subtypes, α-, β-, δ-, ε- and PP-cells, responsible for the production of the hormones glucagon, insulin, somatostatin, ghrelin and pancreatic polypeptide (PP), respectively [1]–[5]. Type 1 Diabetes Mellitus (T1DM) is caused by an autoimmune reaction leading to the loss of insulin-producing β-cells resulting in chronic hyperglycemia. Current therapies allow a measure of control of blood glucose levels in T1DM patients. However, due to the difficulties encountered in maintaining appropriate glycemic levels, such patients exhibit an increased risk of vascular complications [6]. Thus, in an effort to design improved protocols, current research efforts aim at elucidating the genetic programs underlying pancreatic endocrine cell lineage determination and/or reprogramming, an approach that could be of interest for the development of alternative therapies for T1DM. Pancreatic development involves an elaborate process of morphological events accompanied by a complex pattern of cellular differentiation and lineage selection. These events are mediated in great part by tissue interactions, signaling pathways and directed cascades of gene expression that determine cell fate [7], [8]. Thus, Pdx1 plays a critical role in early pancreas development [9]–[13], whereas the bHLH-containing transcription factor Neurogenin 3 (Ngn3) specifies the endocrine cell lineage [14]–[18]. Subsequent endocrine cell fate allocation is largely dependent on the interplay between additional transcription factors, including Arx and Pax4. These were shown to display opposing activities in the embryonic processes underlying the specification toward the endocrine subtype destinies: while Pax4 is instrumental for the allocation to the β- and δ-cell lineages, Arx is involved in specifying the α-cell fate [19]–[21]. Subsequent studies demonstrated that the misexpression of *Arx* in adult β-cells induces their conversion into cells exhibiting α- or PP-cell characteristics [22]. Most interestingly, the ectopic expression of *Pax4* in glucagon-expressing cells was found to induce their regeneration and subsequent conversion into cells displaying most of the typical features of β-cells, these being able to counteract the effects of chemically-induced hyperglycemia [23], [24].

Due to (1) the opposing functions of Pax4 and Arx during embryonic development and (2) the persistent expression of *Arx* in glucagon-producing cells, we sought to determine whether the inhibition of *Arx* expression in α-cells could induce their conversion into β-like cells. To this end, we generated different transgenic models allowing the constitutive or conditional inactivation of *Arx* in glucagon-expressing cells. Using lineage tracing, we provide evidence that glucagon-producing cells, even at relatively advanced ages, may be converted into functional β-like cells solely upon *Arx* inactivation. In addition, following the loss of glucagon-producing cells, a cycle of endocrine cell regeneration is initiated whereby glucagon^+^ cells are subsequently acquiring a β-cell phenotype, ultimately leading to islet hypertrophy due to a β-like cell hyperplasia. Interestingly, the further inactivation of *Pax4* in these animals does not impact these processes, suggesting that Arx is the main player in α-cell reprogramming. Most importantly, upon the chemical induction of diabetes, *Arx* mutants display a clear regeneration of β-cells, reversion of diabetes and an extended lifespan compared to controls.

## Results

### The inactivation of *Arx* in α-cells of different ages results in insulin-producing cell hyperplasia

To determine the consequences of the loss of *Arx* in α-cells, we first generated animals allowing the constitutive deletion of *Arx* in all glucagon-producing cells (**Figure S1 Left**) by crossing the ArxcKO mouse line (in which the second exon of the *Arx* gene is flanked by LoxP sites [25]) with Glu-Cre transgenic animals (generated using a transgene composed of the glucagon promoter driving the expression of the phage P1 Cre recombinase [26]). The resulting double transgenics (referred to as Glu-ArxKO) were further crossed with ROSA26-LoxP-Neomycin Resistance-STOP-LoxP-β-gal animals (containing a transgene encompassing the ubiquitous ROSA26 promoter in front of the neomycin resistance gene with STOP codons flanked by LoxP sites and followed by the *β-galactosidase* cDNA [27] - henceforth referred to as Rosa) for lineage tracing purposes.

A number of tests were performed to determine the efficiency of this strategy. First, by combining several immunohistochemical approaches, we analyzed 2 week-old homozygous Glu-Cre::Rosa animals to further verify the efficiency of glucagon-mediated Cre activity (**Figure S2**). A quantitative analysis demonstrated approximately 72±7% of glucagon^+^ β-gal^+^ cells in the pancreas of these animals (**Figure S2A–C**), a result in line with previously published data [24], [26], [28]. Next, age-matched Glu-ArxKO pancreata were assayed for *Arx* expression by immunohistochemistry: our data indicated a loss of *Arx* expression in approximately 67±6% of glucagon-producing cells (**Figure S2G–I**) as compared to ArxcKO or wild-type controls (**Figure S2D–F**), a proportion matching the ratio of Cre^+^ glucagon-expressing cells. Of note, in a number of these Arx^−^ Glucagon^+^ cells, *Pax4* was clearly detected, suggesting that the loss of *Arx* results in the ectopic expression of *Pax4* in α-cells (**Figure S2G–I arrowheads**). Lastly, qPCR was used to quantify the *Arx* transcript content (**Figure S2J**): a comparison of controls versus Glu-ArxKO pancreata outlined a 74% reduction in *Arx* transcripts upon glucagon-mediated *Arx* deficiency, a result in line with cell quantification. Taken together, our results suggest that, in this experimental model, Cre can efficiently lead to the inactivation of *Arx* in glucagon-producing cells.

In a second set of experiments, to develop an animal model permitting the inducible deletion of *Arx* in adult α-cells (**Figure S1 Right**), the ArxcKO mouse line was crossed with the Glu-rtTA transgenic line (containing a transgene composed of the rat *glucagon* promoter [29] upstream of the *reverse tetracycline-dependent transactivator*) and further mated with TetO-Cre animals (whose transgene includes the *Tet operator* upstream of *Cre Recombinase* cDNA [30]). Of note, Glu-rtTA::TetO-Cre were previously characterized [23]. The resulting triple-transgenic mice will henceforth be referred to as IndGlu-ArxKO (inducible Glu-ArxKO). From hereon, mice treated with Doxycycline for *x* months will be referred to as *x*mDox+. In all cases, untreated animals were found phenotypically similar to their wild-type (WT) counterparts; they will be referred to as “Dox−” or “controls”. The combined analysis of Arx-, glucagon-, and Pax4-producing cells in 1mDox+ IndGlu-ArxKO outlined a loss of *Arx* in approximately 87±9% of glucagon-labeled cells, few of which initiated *Pax4* expression (**Figure S3A–F**), indicating that, in this inducible model, *Arx* can be efficiently inactivated in glucagon-producing cells.

**Figure 1:**
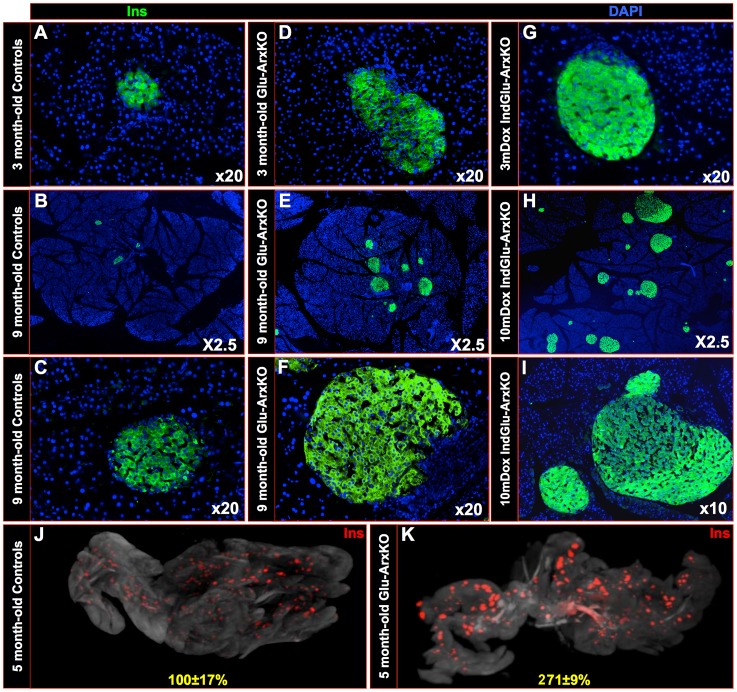
The conditional inactivation of *Arx* in glucagon-producing cells results in islet hypertrophy accompanied by islet neogenesis. (**A–I**) Representative photographs of insulin immunohistochemical analyses performed on pancreata of 3–9 month-old Glu-ArxKO (**D–F**) and on age-/sex-matched controls (**A–C**)). Note the dramatic augmentation in islet size and the substantial increase in islet numbers in mutant animals. Similar alterations were also noted in IndGlu-ArxKO animals treated with Dox for 3 (**G**) and 10 (**H–I**) months. (Each photograph is representative of at least three independent animals). (**J–K**) These results were confirmed by the means of optical projection tomography whereby 5 month-old WT controls (**J**) and age-matched Glu-ArxKO animals (**K**) were assayed for insulin expression (also see **Movie S1**). Quantification of insulin^+^ cell volume revealed a significant 2.71-fold increase in Glu-ArxKO pancreata compared to controls.

Both Glu-ArxKO and Dox+ IndGlu-ArxKO were found viable and fertile, their life expectancy and basal glycemia remaining within normal range (**Table S1-S2**). Interestingly, a substantial increase in islet size was noted in both animal models (**Table S1-S2**). Further analyses indicated that a large insulin^+^ cell hyperplasia was the reason underlying the observed islet hypertrophy (**Figure 1D–I** **, as compared to controls in** **Figure 1A–C**). In Glu-ArxKO animals, a correlation between age and islet overgrowth was apparent, albeit a plateau phase was observed following 4 months of age (**Table S1**). Similarly, in Dox+ IndGlu-ArxKO, the degree of islet hyperplasia was found to depend on the duration of Dox treatment rather than on the age of Dox induction (**Table S2**). As important was the finding that, in both cases, a dramatic increase in islet number was apparent, suggestive of islet neogenesis (**Table S1-S2**). The increased number of islets and the insulin^+^ cell hyperplasia were further demonstrated by means of optical projection tomography allowing the examination of insulin^+^ cells in the entire pancreas (**Figure 1J–K** **, Movie S1**). Indeed, a global pancreatic increase of 171±9% in the number of insulin^+^ cells was thereby outlined in 5 month-old Glu-ArxKO pancreata as compared to age-matched controls. Taken together, our data suggest that the inactivation of *Arx* in adult α-cells (even of relatively advanced ages) results in a clear islet hypertrophy caused by an insulin^+^ cell hyperplasia and a substantial increase in islet number.

### Insulin^+^ cells in *Arx* mutants display a β-cell phenotype

**Figure 2:**
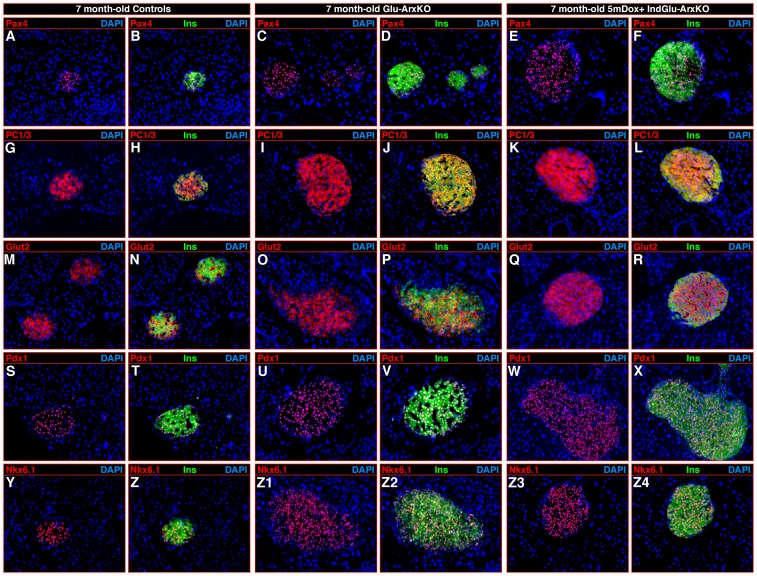
The loss of *Arx* triggered in glucagon-producing cells induces an insulin^+^ cell hyperplasia, such cells expressing *bona fide* β-cell marker genes. Representative photographs of immunohistochemical analyses performed on pancreas sections of 7 month-old WT controls (**A–B, G–H, M–N, S–T, Y–Z**) and on age-/sex-matched Glu-ArxKO (**C–D, I–J, O–P, U–V, Z1–Z2**), as well as on IndGlu-ArxKO mice treated with Dox for 5 months (**E–F, K–L, Q–R, W–X, Z3–Z4**) using the indicated antibody combinations. Compared to controls, both mutant pancreata displayed an increase in islet size caused by an insulin^+^ cell hyperplasia, such cells uniformly expressing the *bona fide* β-cell markers Pax4 (**A–F**), PC1/3 (**G–L**), Glut-2 (**M–R**), Pdx1 (**S–X**), Nkx6.1 (**Y–Z4**), but also MafA, NeuroD1, HB9, and Pax6 (**Figure S4**). (Each photograph is representative of at least three independent animals).

To ascertain the identity of the insulin^+^ cells in Glu-ArxKO and Dox+ IndGlu-ArxKO pancreata, we assayed the expression of several endocrine cell marker genes. Our analyses demonstrated that all insulin^+^ cells observed in Glu-ArxKO and Dox+ IndGlu-ArxKO pancreata expressed the *bona fide* β-cell labels Pax4 **(** **Figure 2A–F**), PC1/3 (**Figure 2G–L**), Glut-2 (**Figure 2M–R**), Pdx1 (**Figure 2S–X**), Nkx6.1 (**Figure 2Y–Z4**), MafA (**Figure S4A–F**), NeuroD1 (**Figure S4G–L**), HB9 (**Figure S4M–R**), and the pan-endocrine marker Pax6 (**Figure S4S–X**). These were found to lack the α-cell determinants, such as Brn-4 and glucagon, as well as somatostatin or PP (see hereafter and data not shown).

**Figure 3:**
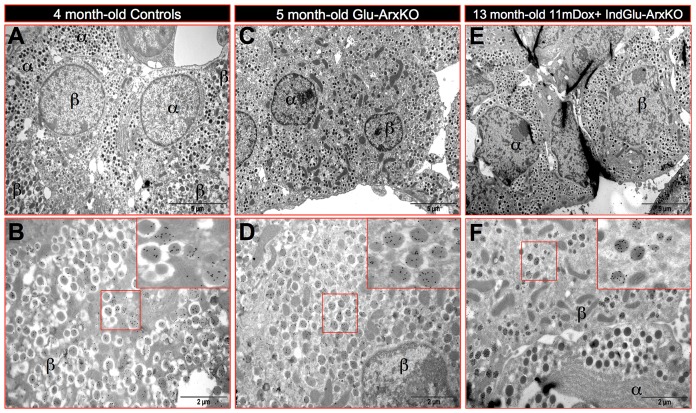
Ultrastructural analysis of Glu-ArxKO and Dox+ IndGlu-ArxKO pancreata by electron microscopy. Classical electron microscopy examination was combined to insulin detection using immuno-gold labeling to analyze islets of 4 month-old WT (**A–B**), 5 month-old Glu-ArxKO (**C–D**) and 11mDox+ IndGlu-ArxKO (**E–F**) animals. In mutant animals, all cells displaying a β-cell ultrastructure expressed insulin (inlets in **D–F** compared to **B**). Similarly, all cells labeled with insulin exhibited a typical β-cell ultrastructure (n = 3, 200 photographs analyzed per sample).

**Figure 4:**
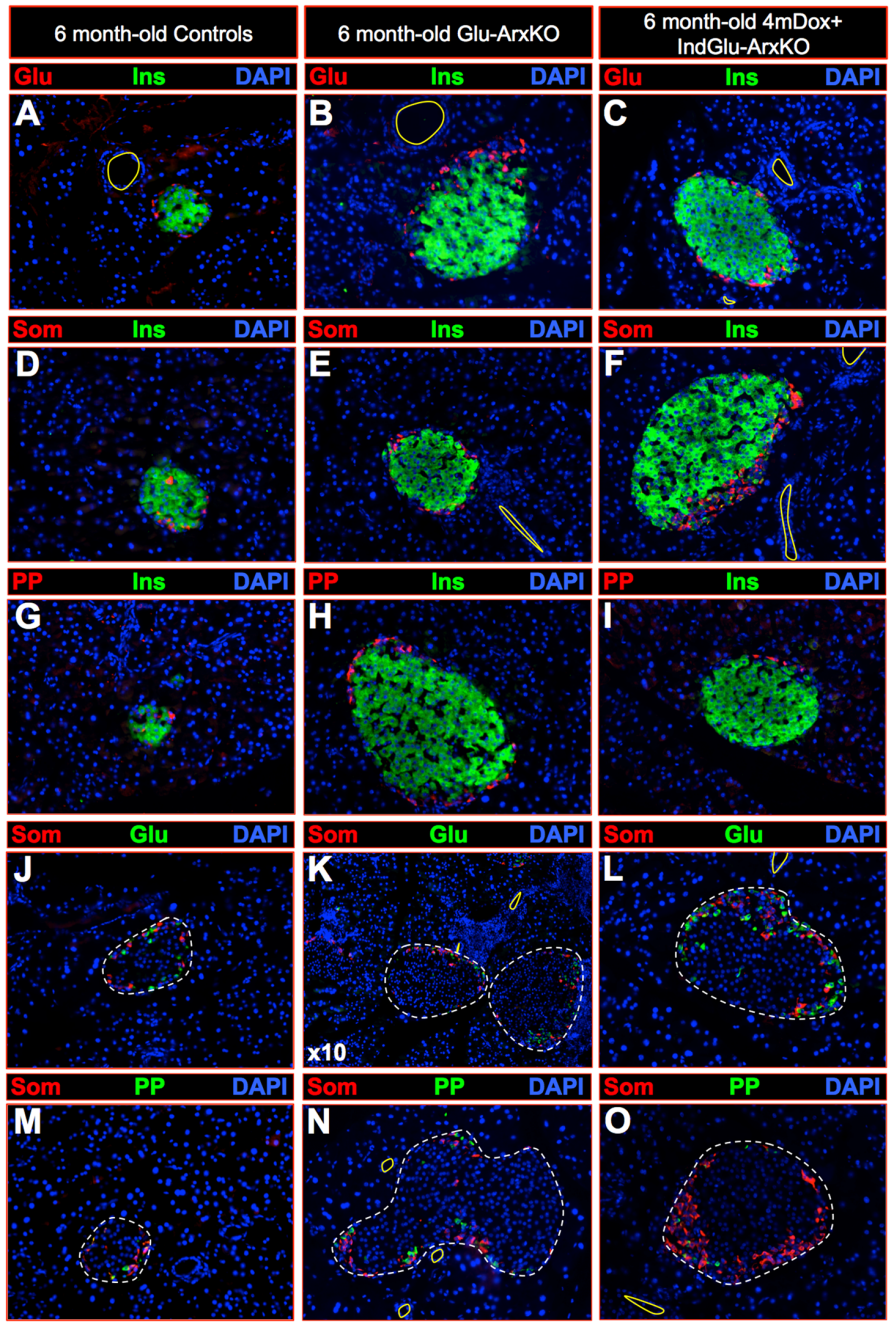
*Arx* inactivation in glucagon-producing cells results in the misallocation of non-β-cells within the islet. Representative photographs of immunohistochemical analyses performed on sections of 6 month-old controls (**A, D, G, J, M**), 6 month-old Glu-ArxKO (**B, E, H, K, N**) and 6 month-old 4mDox+ IndGlu-ArxKO (**C, F, I, L, O**) pancreata using the indicated antibody combinations. Both Glu-ArKO and Dox+ IndGlu-ArxKO animals displayed increased insulin^+^ cell contents compared to controls, these cells being negative for glucagon (**A–C, J–L**), somatostatin (**D–F, J–O**) and PP (**G–I, M–O**). Interestingly, unlike in controls where non-β-cells were distributed within the islet mantle zone (**J, M**), these were found preferentially located at a pole of the islet, adjacent to neighboring ducts in mutants (**K–L, N–O**). For the purpose of clarity, in selected photographs, the ductal lumens are outlined in yellow and islets in dashed white lines.

Since these results suggested that the insulin^+^ cells observed in Glu-ArxKO and Dox+ IndGlu-ArxKO pancreata displayed a β-cell phenotype, we sought to ascertain these observations using electron microscopy combined with insulin detection by immuno-gold labeling. Following the examination of more than 200 sections of pancreas per animal, it appeared that, in both genotypes, all insulin-producing cells displayed a β-cell ultrastructure (**Figure 3C–F** compared to **A–B**). Similarly, all cells displaying a β-cell ultrastructure were found to be positive for insulin. Further analyses of endocrine cells in Glu-ArxKO and Dox+ IndGlu-ArxKO outlined a majority of glucagon^+^ (**Figure 4A–C, J–L**), somatostatin^+^ (**Figure 4D–F, J–O**) or PP^+^ (**Figure 4G–I, M–O**) cells abnormally located at a pole of the islet, close to adjacent ducts. Interestingly, quantitative experiments indicated an increase in the number of cells expressing insulin or somatostatin (**Figure S5A, C**) and variations in the content of glucagon-producing cells (**Figure S5B**). Altogether, our data indicate that the deletion of *Arx* in α-cells at any age leads to a substantial increase in the insulin^+^ cell content, such cells displaying most features of true β-cells. In addition, non-β-cells display an abnormal localization within the islet, close to ducts.

### α-cells are converted into β-like cells upon *Arx* inactivation

**Figure 5:**
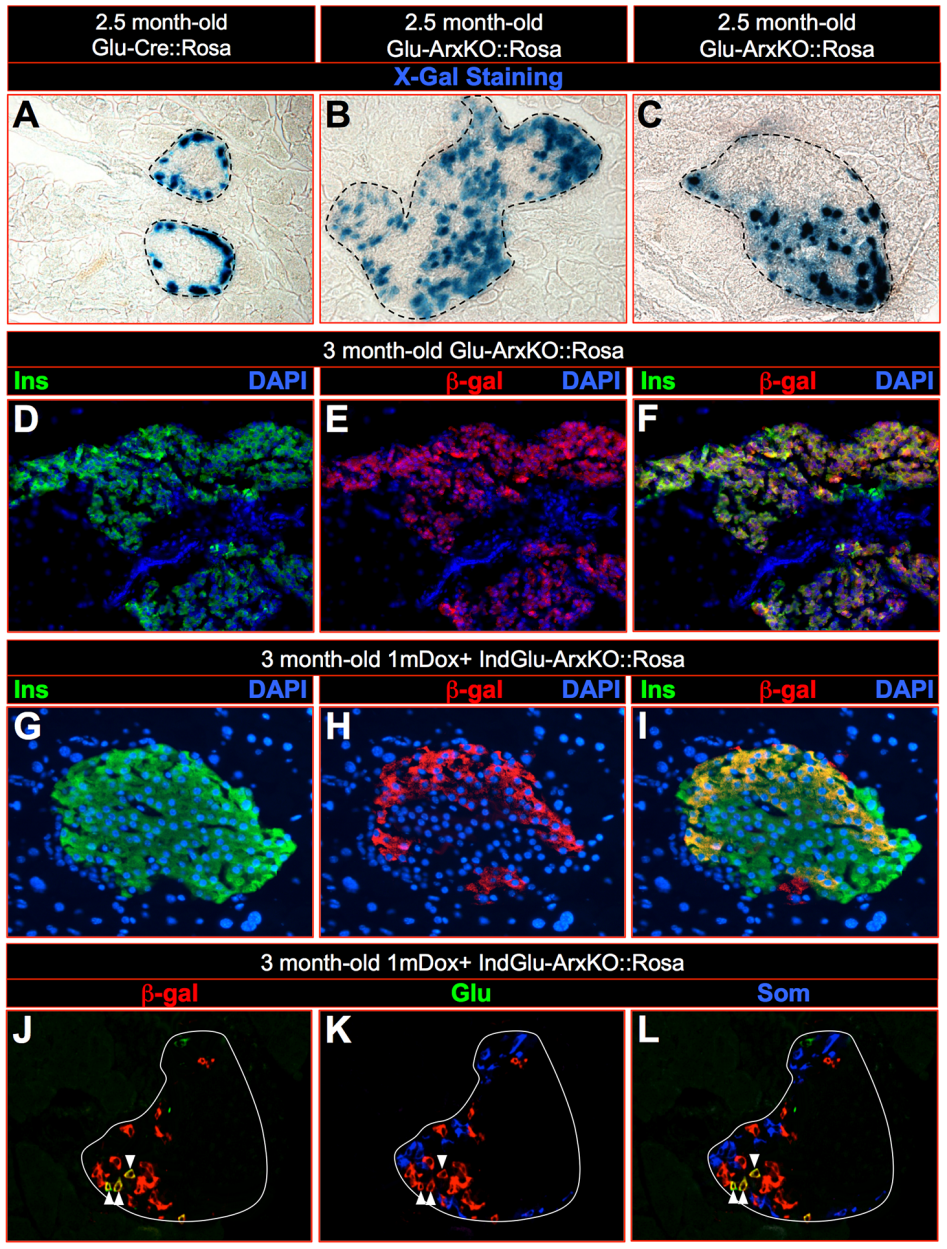
Glucagon-expressing cells can be converted into insulin-expressing cells upon *Arx* inactivation. (**A–C**) By means of X-Gal staining, α-cell lineage tracing on 2.5 month-old Glu-ArxKO::Rosa pancreata showed β-galactosidase activity in a number of cells located within the islet core where insulin^+^ cells are normally located (**B–C**), while β-galactosidase was expectedly and solely detected in the islet mantle of Glu-Cre::Rosa controls (**A**). (**D–L**) Immunohistochemical analyses revealed that most β-gal^+^ cells were in fact insulin^+^ (**D–F**), indicating a conversion of glucagon^+^ cells into insulin^+^ cells. Such a shift in identity was also observed in 1mDox+ IndGlu-ArxKO animals (**G–I**), suggesting that adult/mature α-cells can also acquire a β-like cell phenotype. Interestingly, β-gal labeling was found in insulin^+^ (**G–I**) and glucagon^+^ (**J–L**) cells, but not in somatostatin^+^ cells (**J–L**), demonstrating that these did not pass through a phase of *glucagon* expression. For the purpose of clarity, islets are outlined with dashed black lines in **A–C** or with white lines in **J–L**. In **J–L**, arrowheads provide examples of β-gal^+^ glucagon^+^ cells. (Each photograph is representative of at least three independent animals).

To further characterize the effects of the inactivation of *Arx* in α-cells, we sought to trace their lineage through the analysis of Glu-ArxKO::Rosa pancreata combining X-Gal staining and immunohistochemical approaches (**Figure 5A–F**). In Glu-Cre::Rosa control pancreata, β-gal-labeled cells were solely found in the mantle zone of the islets where glucagon^+^ cells typically reside (**Figure 5A**). Importantly, in Glu-ArxKO::Rosa pancreata, such labeling was also found within the islet core where insulin^+^ cells are classically detected (**Figure 5B–C**). By means of immunohistochemistry, a clear expression of the β-galactosidase enzyme was noted in numerous insulin-producing cells (**Figure 5D–F**), demonstrating a conversion of glucagon-producing cells into insulin-expressing cells. Interestingly, a similar analysis performed in IndGlu-ArxKO::Rosa animals treated with Dox for one month also outlined numerous insulin^+^ β-gal^+^ cells (**Figure 5G–I**). A quantitative analysis revealed that approximately 30 to 40% of neo-generated β-like cells passed through a glucagon-expressing transition phase in both models (**Table S3**). While impressive, this contribution is lower compared to the initial labeling of α-cells (approximately 70 to 80% - **Figure S2, S3**), suggesting the involvement of additional processes. Of note, the co-detection of β-gal with glucagon or somatostatin expectedly revealed glucagon^+^ β-gal^+^ cells while all somatostatin^+^ cells were found negative for β-gal (**Figure 5J–L**), suggesting that the supplementary somatostatin^+^ cells do not derive from cells having expressed the glucagon hormone. Together, our results provide conclusive evidence that α-cells of different ages can be converted into insulin^+^ cells upon the sole inactivation of the *Arx* gene.

**Figure 6:**
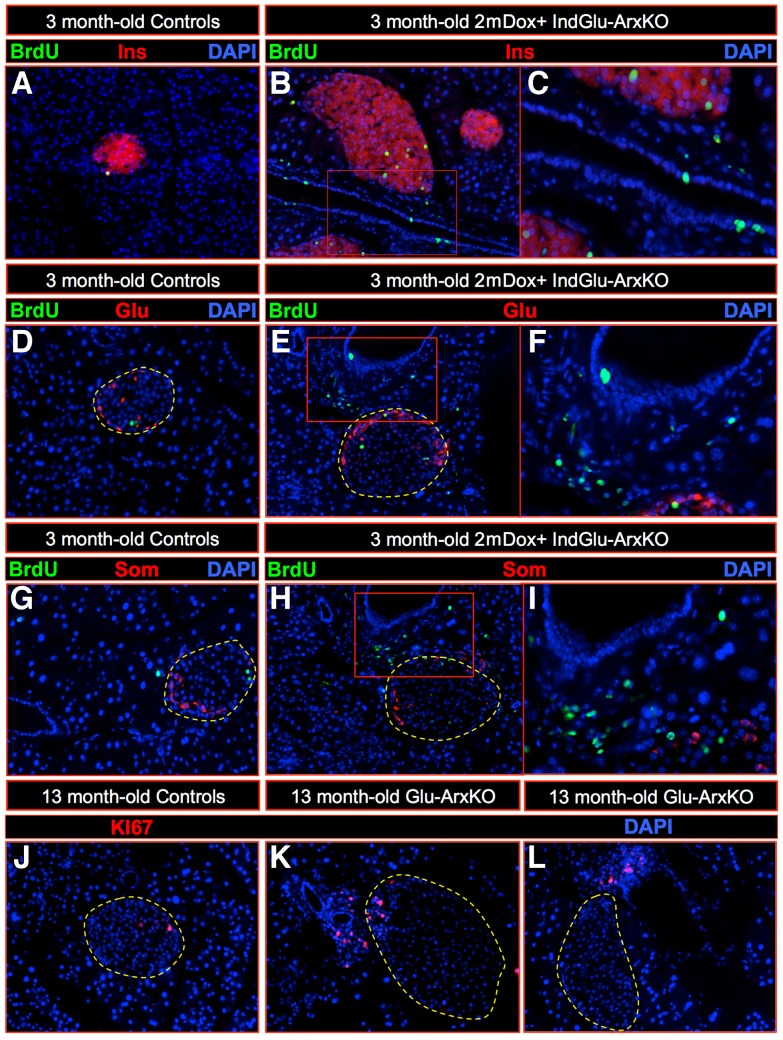
Localized cellular proliferation in the pancreatic ductal lining upon α-cell-specific *Arx* loss. (**A–I**) A 10-day BrdU pulse-chase was performed in 3 month-old IndGlu-ArxKO animals subjected to a 2 month-long Dox treatment. An increase in the number of proliferating cells was thereby noted, most of these being detected in the lining of the duct close to the non-β-cell cluster (**B–C, E–F, H–I** compared to **A, D, G**). Of note, the ductal epithelium was found thickened in mutant pancreata, suggesting an increased ductal cell number (**C, F, I**). (**J–L**) The investigation of cell proliferation in 13 month-old Glu-ArxKO animals also revealed increased numbers of proliferating cells (**K–L**) compared to controls (**J**), as assayed by the expression of the proliferation marker KI67. These were again mainly found in the ductal lining adjacent to the islets (**K–L**). For the purpose of clarity, islets are outlined in dashed yellow lines in selected photographs.

**Figure 7:**
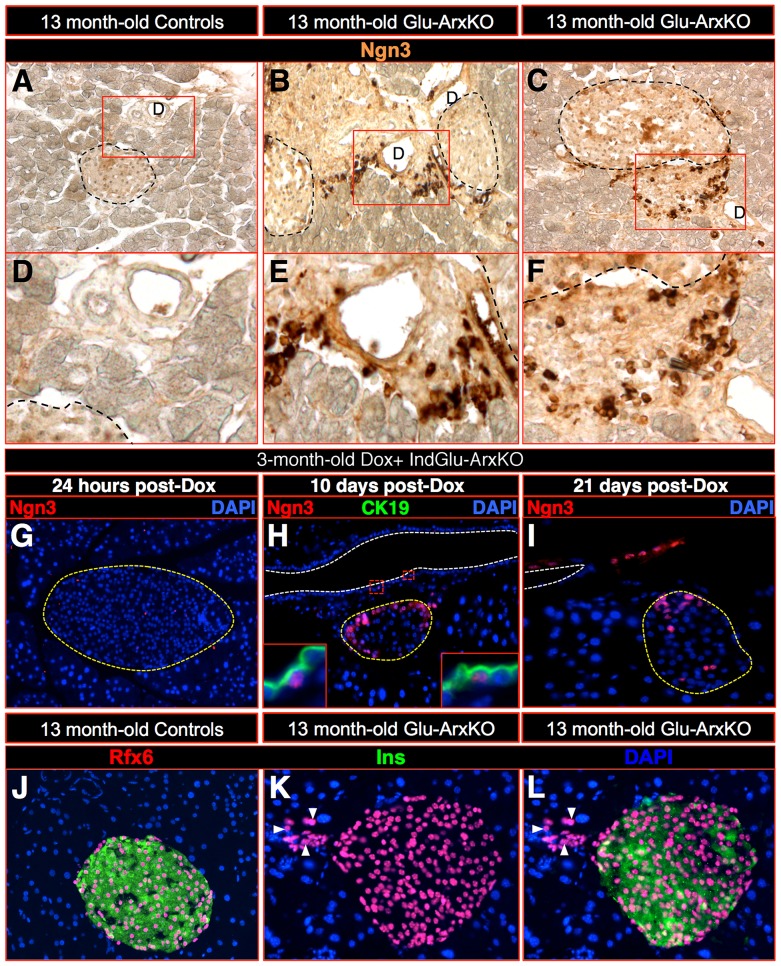
Re-expression of the pro-endocrine factor Ngn3 in the pancreatic ductal lining upon α-cell-specific *Arx* loss. (**A–F**) The investigation of the expression of the pro-endocrine marker *Ngn3* in animals as old as 13 month-old Glu-ArxKO animals revealed its re-expression in the ductal lining of mutant animals (**B–C, E–F**), while being absent or very weakly expressed in their control counterparts (**A, D**). (**G–I**) A time course analysis of Dox+ IndGlu-ArxKO pancreata from animals treated with Dox for increasing durations outlined Ngn3^+^ cells within the ductal epithelium (**H inlet**) and lining (**H**), as well as in islet cells adjacent to ducts, as early as 10 days post Dox treatment initiation (**H** compared to **G**). A similar trend was also noted 11 days later (**I**). The downstream target of Ngn3, Rfx6, classically labeling endocrine cells (**J**), appeared additionally ectopically expressed in the ductal lining of 13 month-old Glu-ArxKO animals (**K–L** - arrowheads outline a few examples). Note that **D–F** photographs correspond to blowups of **A–C**. For the purpose of clarity, in selected photographs, islets are outlined in dashed yellow/black lines while the ductal lumen is outlined in white lines (D: ductal lumen).

**Figure 8:**
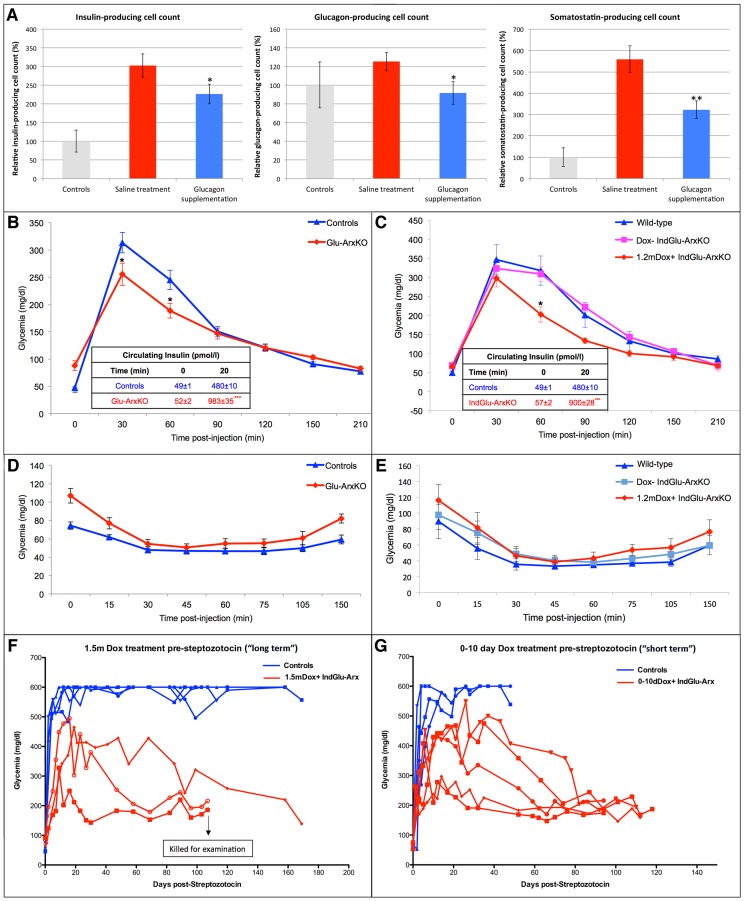
The insulin^+^ cells generated upon *Arx* inactivation are functional. (**A**) To determine the impact of the glucagon shortage provoked by the conversion of α-cells into β-like cells, 2.5 month-old IndGlu-ArxKO animals were supplemented (or not) with exogenous glucagon for 3 weeks as well as with Dox. While saline-treated animals developed significant insulin^+^ and somatostatin^+^ cell hyperplasia, as compared to controls, their glucagon-supplemented counterparts exhibited a diminished increase in insulin^+^ and somatostatin^+^ cell counts accompanied by a decrease in glucagon^+^ cell number. n = 3 ** p<0.01, * p<0.07 using ANOVA comparing saline- and glucagon-treated animals. (**B–G**) 2.5 month-old Glu-ArxKO (and age/sex-matched controls) were subjected to an intraperitoneal glucose tolerance test (**B**). Mutant animals displayed an increased capacity to counteract the glucose bolus with a lower peak in glycemia, suggestive of an increased β-cell mass, further indicated by the augmented levels of circulating insulin in Glu-ArxKO animals compared to their WT counterparts (**Table inserted in B**). Similar results were also evident in 1.2mDox+ IndGlu-ArxKO animals (**C and Table inserted**), where a faster reduction in glucose levels and return to euglycemia were observed compared to both WT and Dox- controls (**C**). The challenge of both transgenic lines with insulin resulted in no significant difference compared to their control counterparts (**D–E**), indicating that insulin remains fully active despite the β-like cell hyperplasia. IndGlu-ArxKO animals were subsequently subjected to streptozotocin treatment after 1.5 months (**F**) or, only 0–10 days (**G**) following Dox treatment initiation. In both cases, by the monitoring of glycemic levels, following an initial peak in glycemia, a steady recovery was noted in the induced animals, while controls either maintained their hyperglycemic state or succumbed to excessive glycemic levels. n>6 for all experiments *** p<0.001, * p<0.05 using ANOVA.

While the conversion of α-cells into β-like cells is of interest, it cannot account for the dramatic β-like cell hyperplasia noted in our animal models, nor for the continuous detection of glucagon^+^ cells despite their conversion. Hence, to gain further insight into the mechanisms underlying these processes, we assayed proliferating cells combining BrdU labeling and immunohistochemical detection of the KI67 marker. Importantly, in 2mDox+ IndGlu-ArxKO pancreata of animals subjected to a long-term 10-day BrdU pulse-chase, we observed a thickened ductal epithelium (**insets in** **Figure 6B–C, E–F, H–I**), most of the BrdU^+^ cells being detected in some epithelial cells and in the adjacent ductal lining while a few appeared located at a pole of the islet where non-β-cells are detected (**Figure 6A–I**). Similar observations were made using KI67 labeling in Glu-ArxKO animals, even in mice as old as 13 months of age (**Figure 6J–L**). We therefore investigated whether *Ngn3* could be re-expressed in animals lacking *Arx* in α-cells. Indeed, *Ngn3* was previously found to be re-expressed in duct-lining cells in animals that underwent pancreatic duct ligation [31], [32] or with the forced misexpression of *Pax4* in α-cells [23], [24]. Additional work suggested that Ngn3^+^ cells could be continuously generated and converted into endocrine cells [23], [24], [32]. Hence, we assayed Glu-ArxKO pancreata for Ngn3 using immunohistochemistry. *Ngn3* has been previously detected in endocrine cells being expressed at very low levels [33]: we confirmed these observations (**Figure 7A, D**). However, a much stronger expression of *Ngn3* was noted mainly in cells located in the pancreatic ductal lining of animals lacking *Arx* in α-cells (**Figure 7B–C, E–F**). A time course analysis of Ngn3^+^ cells in pancreata of IndGlu-ArxKO animals subjected to increasing durations of Dox treatment outlined Ngn3^+^ cells within the ductal epithelium and lining, as well as in islet cells located close to ducts, as early as 10 days post-Dox treatment initiation, a similar trend being detected 11 days later (**Figure 7G–I**). Importantly, the downstream target of Ngn3, Rfx6 [34], was also found ectopically expressed in the ductal lining (**Figure 7J–L**) of Glu-ArxKO animals. These results suggesting a reactivation of, at least, a part of the endocrine differentiation program, we queried the mechanisms triggering such processes. One possibility could be the loss of α-cells, and the resulting shortage in glucagon, provoked by their conversion into β-like cells. To verify this hypothesis, we initiated long-term glucagon supplementation experiments in IndGlu-ArxKO animals. Interestingly, while Dox+ IndGlu-ArxKO pancreata showed a significant augmentation of the insulin^+^ or somatostatin^+^ cells mass following 3 weeks of treatment with saline and Dox (as compared to controls – **Figure 8A**), animals treated with glucagon and Dox exhibited a diminished increase in insulin^+^, glucagon^+^, and somatostatin^+^ cell counts (**Figure 8A**). Taken together, these results provide evidence that, upon α-cell-mediated *Arx* deficiency, α-cells are converted into β-like cells. This, in turn, induces glucagon shortage-dependent regeneration processes characterized by the continuous proliferation of duct-lining cells, the re-expression of the developmental factors Ngn3 and Rfx6, and the compensatory neogenesis of endocrine cells, neo-formed glucagon^+^ cells acquiring a β-like cell identity upon *glucagon* expression and subsequent *Arx* inactivation.

### The sole inactivation of *Arx* can induce the regeneration of a functional β-like cell mass following chemically-induced diabetes

In a continuous effort to ascertain the identity of the supplementary insulin^+^ cells in animals with an α-cell-specific inactivation of *Arx*, numerous tests were performed. It is important to reiterate that both Glu-ArxKO and Dox+ IndGlu-ArxKO animals exhibited normal basal glycemia levels (**Tables S1-S2**). However, upon intraperitoneal glucose tolerance test, these were found to display an improved response with a lower rise in glycemia as compared to control animals (**Figure 8B–C**). This suggests an improved capacity to release insulin upon glucose stimulation. Indeed, when we combined such glucose challenges with insulin measurements, while the basal insulin levels were found within normal range, a dramatic increase in circulating insulin content was noted when compared to controls (**Figure 8B–C** **Tables**). Our results therefore support the notion of an increased β-like cell mass able to respond to a glucose bolus. Importantly, upon insulin challenge and despite the augmented numbers of β-like cells, the resulting glycemic level variations were found similar in Glu-ArxKO, Dox+ IndGlu-ArxKO, and control animals (**Figure 8D–E**). This indicates that, despite a β-like cell hyperplasia, such animals do not exhibit any insulin resistance.

To determine whether neo-formed insulin^+^ cells could functionally replace endogenous β-cells, we injected IndGlu-ArxKO animals with a high dose of streptozotocin to obliterate the endogenous β-cell mass. Two groups of IndGlu-ArxKO animals, and matching controls, were used: the first (“long pre-treatment”) corresponding to animals for which Dox treatment was initiated 1.5 months prior to, and the second (“short pre-treatment”) 0 to 10 days prior to, streptozotocin administration. Dox treatment was continued for both groups until examination (**Figure 8F**–**G** **, respectively**). All control animals developed extreme hyperglycemia leading to the death of the majority of these (**Figure 8F**–**G**). Interestingly, all Dox+ IndGlu-ArxKO animals also developed a strong hyperglycemia but none died (**Figure 8F**–**G**). As important was the observation that, following this hyperglycemic phase, a progressive return to normal levels was outlined (**Figure 8F**–**G**). It is interesting to note that a return to euglycemia was also evident when Dox-mediated *Arx* inactivation in α-cells was triggered as late as on the day of streptozotocin injection (**Figure 8G**), suggestive of efficient/rapid mechanisms allowing the restoration of an appropriate glycemic balance.

**Figure 9:**
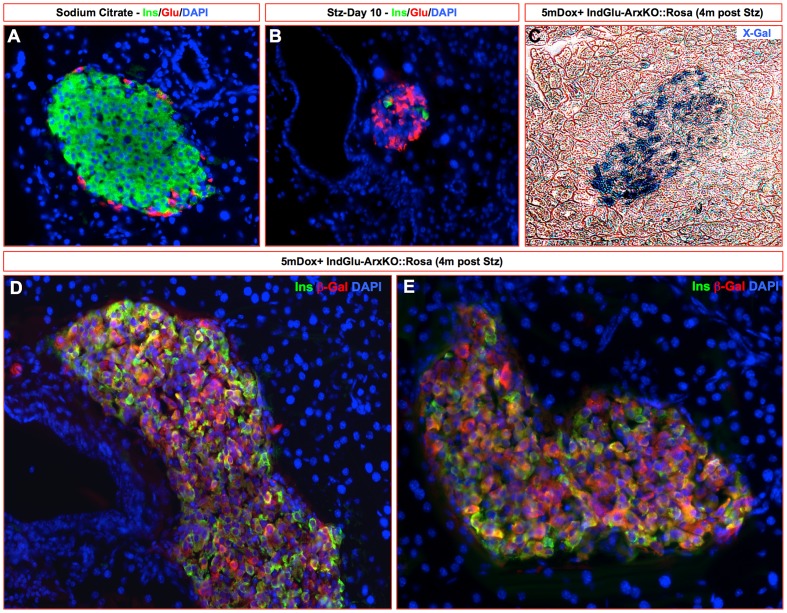
The sole inactivation of *Arx* in α-cells can induce a regeneration of the β-like cell mass following streptozotocin treatment. 1.5mDox+ IndGlu-ArxKO::Rosa animals were treated with streptozotocin or sodium citrate (**A**) and their pancreatic composition was examined by immunohistochemistry. Next to the classical loss of β-cells upon high-dose streptozotocin treatment (data not shown), few insulin-producing cells could be detected as early as 10 days post-streptozotocin treatment (**B**). Interestingly, after 100 days of Dox treatment, the islets in streptozotocin-treated animals appeared reformed and displayed β-gal^+^ cells located in the core of the islet (**C**). Further examination demonstrated that such β-gal^+^ cells were insulin^+^ (**D–E**), suggesting that *Arx* inactivation in glucagon-expressing cells allows their regeneration and conversion to functional insulin^+^ cells capable of counteracting the effects of streptozotocin. (Stz: Streptozotocin, each photograph is representative of at least three independent animals).

By immunohistochemical analyses, we observed a classical loss of β-cells shortly after streptozotocin treatment (**Figure 9A–B** and data not shown). However, a clear neogenesis was outlined in these Dox+ IndGlu-ArxKO animals, resulting in the replenishment of the β-cell mass (**Figure 9B–E**). Importantly, when the same experiments were performed on Dox+ IndGlu-ArxKO::Rosa mice, lineage tracing experiments confirmed that a vast majority of regenerated β-like cells passed through a phase of glucagon expression (**Figure 9C–E**), suggesting glucagon^+^ cell neogenesis prior to the acquisition of a β-like cell identity upon *Arx* inactivation. Taken together, these results further demonstrate that the loss of *Arx* in glucagon-producing cells is sufficient to induce their conversion into β-like cells. Our data also suggest that upon such a conversion, compensatory mechanisms are activated and lead to the neogenesis of endocrine cells among which glucagon^+^ cells are again converted into β-like cells upon *Arx* inactivation. Importantly, this cycle of β-like cell regeneration is able to counter toxin-induced diabetes, such cells being able to maintain euglycemia.

### The additional inactivation of *Pax4* in *Arx* mutants does not impact the α-cell-mediated β-like cell neogenesis

**Figure 10:**
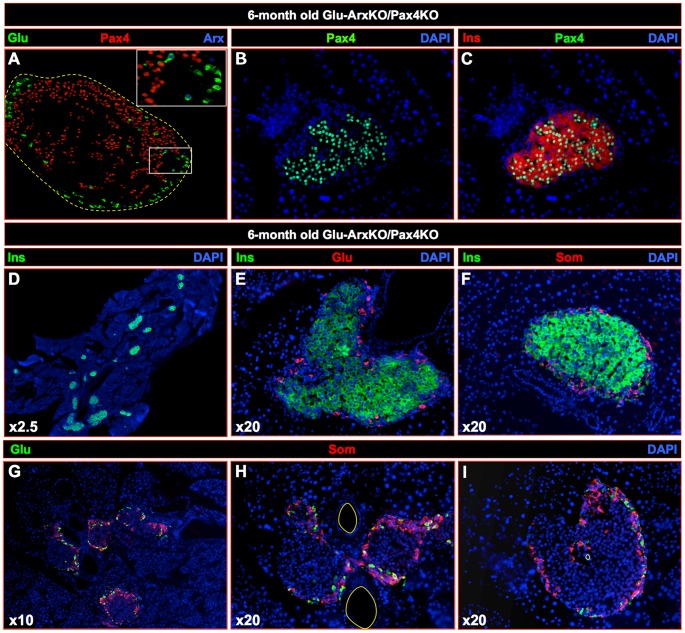
The inactivation of *Pax4* in *Arx*-deficient glucagon-expressing cells does not impact β-like cell neogenesis. Representative photographs of immunohistochemical analyses performed on 6 month-old Glu-ArxKO/Pax4KO pancreata using the indicated antibody combinations. A clear loss of *Arx* was evidenced in Glu-ArxKO/Pax4KO glucagon^+^ cells (**A**), such cells not ectopically expressing *Pax4* (as seen in **Figures S2-S3**). Interestingly, a number of insulin^+^ cells appeared Pax4^−^, such cells most likely deriving from Arx^−/Y^ Pax4^−/−^ glucagon^+^ cells (**A–C**). As noted in Glu-ArxKO and Dox+ IndGlu-ArxKO pancreata, an increase in islet size compared to controls (**Figure 1**), caused by an insulin^+^ cell hyperplasia, was observed in Glu-ArxKO/Pax4KO pancreata (**A–I**). Non-β-cell endocrine hormone-expressing cells displayed a preferential localization at poles of the islets, adjacent to neighboring ducts (**E–I**), reminiscent of the phenotype of the sole inactivation of *Arx* in glucagon^+^ cells. (Each photograph is representative of at least three independent animals).

**Figure 11:**
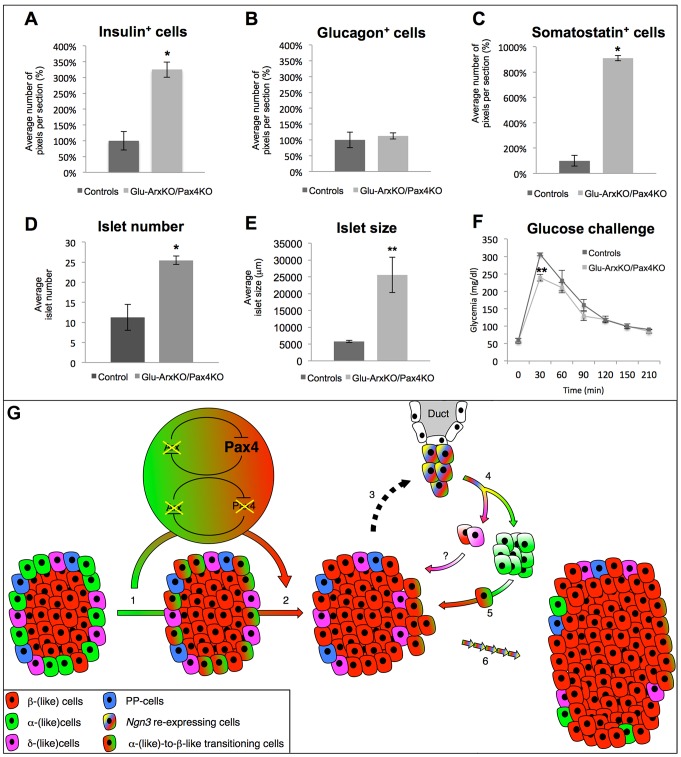
Quantitative analyses upon the dual inactivation of *Arx* and *Pax4* in glucagon-producing cells. (**A–E**) Quantitative comparison of the number of insulin- (**A**), glucagon- (**B**) and somatostatin- (**C**) expressing cells between 4 month-old Glu-ArxKO/Pax4KO animals and age-/sex-matched WT controls. A significant increase in the numbers of insulin- and somatostatin-expressing cells was observed in Glu-ArxKO/Pax4KO animals, whilst no significant variation in glucagon^+^ cells was noted. Interestingly, both islet count (**D**) and size (**E**) were significantly increased in these animals compared to their WT counterparts, suggesting a process of islet neogenesis in addition to an increased insulin^+^ cell mass. (**F**) 4 month-old Glu-ArxKO/Pax4KO animals (and age/sex-matched WT controls) were challenged with glucose. Double-mutant animals displayed an increased capacity to counteract the glucose bolus with a lower peak in glycemia, suggestive of a functional increased β-cell mass. n≥3 in all experiments, ** p<0.01, * p<0.05 using ANOVA. (**G**) Schematic detailing the consequences of *Arx* (and *Pax4*) inactivation triggered in α-cells. Following the inactivation of *Arx* (and *Pax4*), α-cells are converted into β-like cells (1–2). The resulting shortage in glucagon (and/or putative additional signals - 3) promotes the proliferation of duct-lining cells, some of which re-express the developmental factor Ngn3. Our results indicate that such Ngn3^+^ cells adopt an endocrine cell identity, the glucagon^+^ cell fate being clearly favored (4). Whether neo-generated somatostatin^+^ cells contribute to the supplementary β-like cell mass remains to be determined (“?”). Similarly, one could assume that Ngn3^+^ cells could directly give rise to β-like cells, but additional experiments and mouse lines would be required to address this question (“?”). Subsequently, neo-formed glucagon^+^ cells are, yet again, turned into β-like cells upon the inactivation of *Arx* (and *Pax4*) (5). Such repeated cycles of neogenesis/double conversion (3 to 5) eventually result in an islet hypertrophy caused by a β-like cell hyperplasia (6).

It was previously shown that the forced expression of *Pax4* in glucagon^+^ cells was sufficient to induce their neogenesis and conversion into β-like cells [23], [24]. Here, we show that, in fact, the inactivation of *Arx* initiated in embryonic, but also in adult, α-cells is sufficient to induce a similar outcome. One may therefore conclude that the misexpression of *Pax4* in α-cells could induce the down-regulation of *Arx* and thereby trigger α-cell mediated β-like cell neogenesis. However, the opposite could also be true, that is, that the deletion of *Arx* could promote such processes by up-regulating *Pax4*. To discriminate between these two possibilities, we generated double-mutant animals allowing the conditional deletion of *Arx* and *Pax4* specifically in α-cells. To achieve this purpose, we crossed ArxcKO animals with Pax4cKO animals (generated by knock-in of two LoxP sites within the *Pax4* locus [35]). The resulting double transgenic animals were subsequently crossed with Glu-Cre mice to generate Glu-Cre::ArxcKO::Pax4cKO animals (referred to as Glu-ArxKO/Pax4KO). Using immunohistochemistry on 6 month-old triple transgenic pancreata, most glucagon-producing cells were found to be negative for both *Arx* and *Pax4* (**Figure 10A**). Importantly, a number of insulin-producing cells were also found to lack Pax4, such cells most likely corresponding to α-cells converted into Arx^−/Y^ Pax4^−/−^ β-like cells (**Figure 10B–C**). Further examination of these triple transgenic pancreata by immunohistochemistry outlined, yet again, a substantial increase in the islet number and a clear islet hypertrophy caused by an insulin^+^ cell hyperplasia that was found to be similar to the one observed in animals with *Arx* deletion (**Figure 10D** **compared to 1E–H**). Quantitative analyses confirmed this augmentation in insulin^+^ cell numbers (**Figure 11A–E**), but also in the content in somatostatin^+^ cells, non-β-cells being again found preferentially located close to ducts within the islets (**Figure 10E–I**). Of note was the observation that, despite the lack of *Pax4* in a number of β-cells, no alteration in basal glycemia of 4 month-old double-mutants could be detected as compared to controls (127±7 mg/dl and 121±4 mg/dl, respectively). Interestingly, upon glucose challenge, Glu-ArxKO/Pax4KO animals displayed a significantly improved response as previously seen in Glu-ArxKO mice (**Figure 11F**), suggestive of an increased functional β-like cell mass. Altogether, our analyses indicate that the combined loss of *Arx* and *Pax4* in glucagon-producing cells results in a phenotype similar to that of *Arx* mutants, sustaining the notion that *Arx* represents the main player involved in α-cell-mediated β-like cell neogenesis processes.

## Discussion

In this study, we report that the inactivation of the *Arx* gene in pancreatic α-cells at different ages results in hypertrophic islets mainly composed of cells displaying a β-cell phenotype. Our data support the notion of a conversion of α-cells into β-like cells upon the sole inactivation of *Arx*. These processes trigger compensatory mechanisms, associated with the re-expression of Ngn3 and Rfx6 in duct-lining cells, resulting in the neogenesis of endocrine cells among which glucagon^+^ cells are subsequently converted into β-like cells upon *Arx* inactivation. Importantly, the newly-formed β-like cells are functional and can reverse chemically-induced diabetes. As interesting was the finding that the additional loss of *Pax4* does not impact these mechanisms, suggesting that Arx represents the main inducer of α-cell-mediated β-like cell neogenesis.

### *Arx* inactivation induces α-cells to acquire a β-like cell identity

We initially analyzed two transgenic mouse models allowing either (1) the constitutive inactivation of *Arx* in all glucagon-producing cells as soon as they initiate hormone expression (that is, during embryogenesis), or (2) the inducible loss of *Arx* in α-cells at different ages. In both instances, the inactivation of *Arx* was found to be fairly efficient with 70–80% of glucagon^+^ cells appearing *Arx*-deficient. Interestingly, a number of Arx^−/Y^ glucagon^+^ cells were found to ectopically express the β-cell-specific gene *Pax4*. As Pax4 and Arx were previously found to mutually inhibit each other's transcription during the course of pancreas morphogenesis and endocrine cell fate allocation [21], it is conceivable that the loss of *Arx* may result in *Pax4* reactivation in adult α-cells. This would suggest that Arx acts in α-cells to maintain their phenotype through the repression of *Pax4* expression. Supporting this notion were the phenotypic alterations observed in both models reminiscent of those found in animals with the ectopic expression of *Pax4* in glucagon-producing cells [23], [24]. Indeed, a progressive insulin^+^ cell hyperplasia/islet hypertrophy and a substantial increase in islet numbers were noted. However, unlike in animals constitutively misexpressing *Pax4* in glucagon^+^ cells [24], both Glu-ArxKO and Dox+ IndGlu-ArxKO mice displayed a normal life expectancy, a normal basal glycemia, and a restricted increase in overall islet/insulin^+^ cell populations. These discrepancies can most likely be attributed to differences in the efficiency of the transgene expression, but also, in the case of Dox+ IndGlu-ArxKO, to the fact that *Arx* deficiency is triggered in α-cells at older ages. A recent report by Wilcox et al. [36] using a similar approach, and mostly focusing on embryogenesis, is in agreement with the notion of α-to-β-like cell conversion upon *Arx* inactivation, although milder phenotypic alterations were observed, most likely due to low efficiency in Cre-mediated recombination processes. However, an induction of *Arx* inactivation in the adult context did not result in any cell conversion, most likely due to the very short induction times and the use of a non-specific Cre expression system. Herein, using lineage tracing, we demonstrate that the loss of *Arx* in α-cells induces their conversion into cells displaying most features of true β-cells, as outlined through marker gene analyses, electron microscopy examination, and functional assays. Such plasticity of α-cells had been previously suggested using alternative approaches, but never using gene inactivation [24], [37], [38]. It is interesting to note the similarity in phenotypic alterations in Glu-ArxKO mice and IndGlu-ArxKO animals treated with Dox at different ages. Indeed, this indicates that aging is not a limiting factor in this conversion process, or in the resulting insulin^+^ cell neogenesis. In other words, our data demonstrate that adult α-cells, having been subjected to aging and environmental signals, retain their potential for conversion into β-like cells by the selective inhibition of a single gene, *Arx*.

### Origin of the neo-generated β-like cells

While we demonstrated a conversion of glucagon-producing cells into β-like cells, we also consistently observed the continued presence of glucagon^+^ cells in both animal models. In addition, quantification analyses did not show significant variations in their content as compared to controls. Of note, such glucagon^+^ cells were found to be PC1/3^−^ GLP1R^−^, indicating that they display a mature α-like cell phenotype. However, the lack of additional α-cell markers calls for caution concerning the degree of maturity of such glucagon^+^ cells. The continued detection of glucagon^+^ cells suggests that neogenesis processes are activated in order to compensate for the loss of the converted α-cells. Accordingly, the glucagon shortage provoked by this conversion was found to contribute to this neogenesis, as seen in animals misexpressing *Pax4* in glucagon^+^ cells [24]. Interestingly, numerous proliferating cells were observed in *Arx* mutant pancreata. These were mostly detected in the ductal lining instead of within the islet. In addition, cells re-activating the expression of the developmental and pro-endocrine gene *Ngn3* were also detected in the same location as seen in animals that underwent pancreatic duct ligation [31], [32] or with forced misexpression of *Pax4* in α-cells [23], [24]. Along the same line, an Ngn3 target, Rfx6, was also found ectopically expressed in the same location, suggesting a recapitulation of, at least, a part of the endocrine differentiation program. Whether known additional developmental targets of Ngn3, such as IA1 or Myt1, are also reactivated is a challenging question. Indeed, Ngn3^+^ cells appear to quickly migrate from the ductal compartment to the islet in which most endocrine cells are positive for IA1 or Myt1. Thus, while our data support the notion of the existence of a putative pancreatic precursor cell niche, the exact nature and potential of the *Ngn3* re-expressing cells remains to be determined. Such cells do not appear to be stem-like cells as, using qPCR, we could not detect any variation in the transcripts of the classical stem-cell markers genes (such as Oct4 or Nanog - data not shown). However, our results suggest that such Ngn3^+^ cells have the potential to adopt any of the four endocrine cell lineages. Altogether (see model in **Figure 11G**), our analysis of α-cell-specific *Arx* mutants supports the notion that, upon the conversion of α-cells into β-like cells, compensatory mechanisms are activated and result in the proliferation of duct-lining cells, the re-expression of *Ngn3*, as well as the ectopic expression of *Rfx6*, and the acquisition of an endocrine cell identity. Among these neo-generated islet cells, glucagon^+^ cells are further converted into β-like cells upon *Arx* inactivation, such a cycle resulting into β-like cell hyperplasia. The validation of this concept would require the inducible tracing of the lineage of *Ngn3*-, *Rfx6*-, *HNF1β*, and/or *Ptf1a*-expressing cells. However, having employed the Cre/Lox system to inactivate *Arx* in these animal models, this strategy could not be used to follow the progeny of these different cell subtypes.

While lineage tracing experiments allowed us to conclude that continuously neo-generated glucagon^+^ cells can be converted into β-like cells upon *Arx* inactivation, the initial 70–80% labeling of α-cells did not fully match the subsequent 30–40% marking of β-like cells. One could therefore ponder the origin of neo-generated β-gal^−^ supplementary β-like cells. As *Ngn3* is re-expressed in cells of the ductal lining and since some of the endocrine cell developmental pathways appear to be reactivated, one could expect Ngn3^+^ cells to mainly adopt a β-like cell fate as seen during pancreas morphogenesis, such cells would thus be β-gal^−^. Another possibility concerns somatostatin^+^ cells. Indeed, we observed a large increase in their number in both animal models, such an increase not progressing over time; thus, one could hypothesize a continuous neogenesis of these cells prior to a putative conversion into β-like cells, as seen with glucagon^+^ cells. Lastly, one could also hypothesize a contribution of acinar cells as elegantly demonstrated by Pan *et al*. using *Ptf1a* lineage tracing in animals that underwent pancreatic duct ligation [31]. As mentioned previously, the determination of the exact mechanisms involved would be complicated as the tracing of Ngn3^+^/HNF1β^+^/Ptf1a^+^ cells would, yet again, be required. Similarly, tracing somatostatin^+^ cells is, to the best of our knowledge, currently impossible as a working Somatostatin-Cre mouse line still remains to be generated. It is worth noting that, upon streptozotocin treatment, a large proportion of the regenerated β-like cells appeared to have transitioned through a glucagon-expressing cell phase, suggesting that additional mechanisms/facultative precursor cells could be mobilized upon such severe β-cell depletion. Yet again, the determination of the exact processes involved will have to await the generation of the proper mouse lines.

### The inactivation of *Arx* in α-cells can reverse the consequences of toxin-induced diabetes

In a continuous effort to ensure the identity of the supplementary β-like cells observed in Glu-ArxKO and Dox+ IndGlu-ArxKO mice, we monitored their physiological parameters and performed functional assays, including glucose and/or insulin level measurements upon glucose or insulin challenges. In all cases, our results support the notion of an increased β-like cell mass capable of responding to different challenges. Of note, despite a β-like cell hyperplasia, these animals were found to exhibit a normal basal glycemia and did not display any insulin resistance, suggestive of a feedback loop allowing optimal regulation. Importantly, our analyses showed that such β-like cells could functionally replace their endogenous counterparts upon streptozotocin-induced β-cell ablation and thereby prevent animal death or chronic hyperglycemia. Interestingly, our data also suggest that such regenerative processes can lead to a replenishment of the β-cell mass, and thereby allow animal survival, even when triggered concomitantly with toxin-mediated hyperglycemia. This indicates a relatively efficient and rapid β-like cell neogenesis. Thus, our findings provide evidence that the sole loss of the *Arx* gene in α-cells is sufficient to initiate a continuous cycle of glucagon^+^ cell neo-formation and their conversion into β-like cells, and thereby lead to the regeneration a functional β-like cell mass, a concept of importance in the context of diabetes research.

### *Arx* as a main player in β-like cell neogenesis

As mentioned previously, in a number of Glu-ArxKO and Dox+ IndGlu-ArxKO glucagon-producing cells, we noted an ectopic expression of *Pax4*. While the apparently rapid α-to-β-like cell conversion did not allow us to observe this misexpression in all Arx^−/Y^ glucagon^+^ cells, one could hypothesize that *Pax4* re-expression in α-cells could, at least in part, contribute to their conversion into β-like cells as previously reported [24]. Thus, to conclusively determine whether the forced expression of *Pax4* or the inactivation of *Arx* is at the origin of the conversion of glucagon^+^ cells into β-like cells, we generated an animal model allowing the inactivation of both genes in glucagon^+^ cells. Interestingly, we observed phenotypic alterations similar to that of *Arx*-deficient or *Pax4*-misexpressing animals [24], including an insulin^+^ cell hyperplasia, an augmentation in the islet number, and a preferential location of non-β-cells close to ducts. It is worth noting that a number of Pax4^−^ insulin^+^ cells were observed in these double-mutant pancreata, providing information on their origin and thereby indicating that these were derived from glucagon-producing cells. Thus, these results demonstrate that *Pax4* is dispensable for these regeneration processes and allow us to conclude that *Arx* represents the main factor allowing the conversion of α-cells into β-like cells and their subsequent regeneration. We therefore propose that the development of therapies aiming at inactivating/inhibiting Arx in α-cells could potentially open new avenues for diabetes research and/or aid the design of efficient β-cell differentiation protocols in the context of stem cell research.

## Materials and Methods

### Ethics statement

All mouse work was conducted according to French ethical regulations. This project received the approval from our local ethics comity (NCE/2011-22).

### Mouse manipulations

To characterize the effects of the invalidation of the *Arx* gene in pancreatic glucagon-producing cells, different transgenic lines were used. Firstly, using the Cre-LoxP system, Glu-Cre::ArxcKO animals were generated by the crossing of the previously described ArxcKO mouse line [25], generated by homologous recombination by inserting two LoxP sites around the second exon of the Arx gene, and the classical glucagon-Cre mouse line, described by Herrera et al. [26]. Lineage tracing experiments were achieved by the crossing of Glu-Cre::ArxcKO animals with the ROSA26-lox-stop-lox-β-Gal mouse line [27] to generate Glu-Cre::ArxcKO::ROSA26-β-Gal triple-transgenic mice. Secondly, taking advantage of the TetOn system (Clontech), double transgenic mice obtained by the crossing of Glu-rtTA and TetO-Cre mice [23], were further mated with the ArxcKO::ROSA26-β-Gal mouse line. In the resulting transgenic mouse line, upon Doxycycline treatment, *Arx* is specifically inactivated in glucagon-expressing cells whose lineage can be followed. Note that the analyzed animals were mostly males as *Arx* is located on the X chromosome. For best results, we mostly used animals homozygous for the other transgenes. Doxycycline (Sigma) was administered via the drinking water prepared freshly once a week at a concentration of 2 g/L. To assess cellular proliferation, animals were treated with BrdU (in drinking water) for 10 days prior to examination (1 mg/ml solution). To determine the effects of glucagon supplementation on islet size, mice were injected intraperitoneally twice daily with 5 µg of glucagon (Sigma) for 3 weeks. To analyze the inactivation of *Pax4* in *Arx* mutants, *Pax4* conditional knockout mice, previously described by Kordowich et al. [35], were crossed with Glu-Cre::ArxcKO animals (Glu-Cre::ArxcKO::Pax4cKO).

### Immunohistochemistry

Tissues were fixed in 4% PFA for 30 min at 4°C, embedded in paraffin and 8 µm sections applied to slides. These sections were assayed as described previously [19]. The primary antibodies used were the following: guinea pig polyclonal anti-insulin (1/500-Linco), anti-glucagon (1/500-Linco), mouse monoclonal anti-insulin (1/500-Sigma), anti-glucagon (1/500-Sigma), rat anti-somatostatin (1/250-Millipore), anti-KI67 (1/100-Dako), rabbit anti-PP (1/500-Millipore), anti-Glut2 (1/500-Chemicon), anti-PC1/3 (1/500-Millipore), anti-Nkx6.1 (1/3000, kindly provided by S. Heller), anti-Pdx1 (1/1000, kindly provided by C. Wright), anti-NeuroD1 (1/500-Millipore), anti-Pax4 (1/4000, kindly provided by B. Sosa-Pineda), anti-Arx (1/500, generated in house), anti-HB9 (1/500-Chemicon), anti-β-gal (1/10000-Cappel), anti-MafA (1/500-Abcam). The secondary antibodies (1/1000 - Molecular Probes) used were: 594-alexa anti-mouse; 488-alexa anti-mouse; 594-alexa anti-rabbit; 488-alexa anti-rabbit; 594-alexa anti-guinea pig; 488-alexa anti-guinea pig; 488-alexa anti-rat. Pictures were processed using ZEISS Axioimager Z1 and LEICA DM 6000 B. For quantification purposes, *in silico* pixel counting was used on every tenth section.

### β-galactosidase-based lineage-tracing experiments

Pancreatic tissues were isolated and fixed for 30 min at 4°C in a solution containing 1% formaldehyde, 0.2% glutaraldehyde, 0.02% NP40. The tissues were dehydrated in 25% sucrose overnight at 4°C. Prior to sectioning, tissues were embedded in freezing medium. For β-galactosidase activity assessment, the tissues were washed in PBS and then incubated overnight at 30°C in staining solution (500 mM K_3_Fe(CN)_6_, 250 mM K_4_Fe(CN)_6_, 0,5 M MgCl_2_, 40 mg/ml X-gal in DMF).

### Induction of streptozotocin-mediated diabetes

To induce hyperglycemia, STZ (Sigma) was dissolved in 0.1 M sodium citrate buffer (pH 4.5), and a single dose was administered intraperitoneally (100–200 mg/kg) within 10 min of dissolution. Diabetes progression was assessed by monitoring the blood glucose levels and/or survival rates of mice.

### Challenges and blood glucose levels measurement

For challenge purposes, animals were fasted for 16–18 h and injected intraperitoneally with glucose (2 g/kg of bodyweight) or insulin (0.75 U/kg). Blood glucose levels were measured at the indicated time points post-injection with a ONETOUCH Vita glucometer (Life Scan, Inc., CA).

### RNA analysis

RNA was isolated (RNAeasy, Qiagen) and cDNA synthesis (SuperScript choice system, Invitrogen) was performed according to the manufacturer's instructions. Quantitative RT–PCR was carried out using validated primers (Qiagen) and the QuantiTect SYBR Green RT-PCR Kit (Qiagen) following manufacturer's instructions. PCR reactions and detection were performed on a Mastercycler ep realplex cycler using GAPDH and HPRT1 as internal controls for normalization purposes.

### Electron microscopy

For ultrastructural analyses, anesthetized mice were perfused transcardially with physiological serum, then with 2% glutaraldehyde in 0.1 M cacodylate buffer. Pancreas were dissected, immerged in fixative for hours, post-fixed for 2 h in 1% osmium tetroxide in 0.1 M cacodylate buffer and embedded in epoxy resin. Contrasted ultrathin sections (70 nm) were analyzed under a JEOL 1400 transmission electron microscope mounted with a Morada Olympus CCD camera.

For immuno-gold staining, 200 islets, isolated by collagenase (1 mg/ml) digestion, were fixed with 4% paraformaldehyde, 0.2% glutaraldehyde in 0.1 M phosphate buffer (PB) (pH 7.4) overnight at 4°C and were processed for ultracryomicrotomy according to a slightly modified Tokuyasu method [39]. Immunostainings were processed with an automated immuno-gold labeling system Leica EM IGL using guinea-pig anti-insulin primary antibody (1/500) and 15 nm colloidal gold conjugated protein AG (CMC, University Medical Center, Utrecht, The Netherlands). Sections were examined under a JEOL 1400 transmission electron microscope.

### Optical projection tomography, volumetric quantification and image analysis

OPT analysis was performed as previously described [40], [41]. Each specimen was scanned using the Bioptonics 3001 OPT scanner with a resolution of 1024×1024 pixels and reconstructed with the NRecon version 1.6.1.0 (Skyscan) software. Quantification of the insulin-producing cell mass was undertaken using Imaris software (Biplane). Volumes were calculated by applying a “find objects by intensity” task to select voxels above a specified intensity. The intensity threshold value was manually determined for each image stack. All pancreata was scanned and analyzed blinded.

## Supporting Information

Figure S1 Generation of Glu-ArxKO and IndGlu-ArxKO animals. (**Left**) ArxcKO animals (in which the second exon of the *Arx* gene is flanked by LoxP sites) were crossed with the Glu-Cre mouse line (generated using a transgene composed of the glucagon promoter driving the expression of the phage P1 Cre recombinase). The resulting double transgenics (referred to as Glu-ArxKO) were further crossed with ROSA26-LoxP-Neomycin Resistance-STOP-LoxP-β-gal animals (“Rosa”, containing a transgene encompassing the ubiquitous ROSA26 promoter in front of the neomycin resistance gene with a STOP codon flanked by LoxP sites and followed by the *β-galactosidase* cDNA) for lineage tracing purposes. (**Right**) In a second mating scheme, ArxcKO animals were mated to Glu-rtTA mice (containing a transgene composed of the rat *glucagon* promoter upstream of the *reverse tetracycline-dependent transactivator*) and further mated with TetO-Cre animals (whose transgene includes the *Tet operator* upstream of *Cre Recombinase* cDNA). The resulting triple-transgenic mice, referred to as IndGlu-ArxKO, were subsequently mated with Rosa animals for lineage tracing purposes (IndGlu-ArxKO::Rosa).
(TIF)

Figure S2 Validation of the Glu-ArxKO mouse line. (**A–C**) Aiming to further demonstrate the efficiency of the glucagon-mediated expression of the Cre recombinase, Glu-Cre animals were initially crossed to Rosa mice. Using immunohistochemistry with antibodies raised against glucagon or β-galactosidase, we showed a clear co-expression of the β-galactosidase with the glucagon hormone. In fact, quantitative analyses showed that 72% of glucagon-producing cells were β-galactosidase^+^, further demonstrating the relatively high efficiency of the Cre recombinase expression in this cell subtype. (**D–I**) Pancreata of 2 month-old controls (**D–F**) and of Glu-ArxKO (**G–I**) animals were subjected to quantitative analyses using a co-detection of Arx, Glucagon and Pax4. While in control pancreata, 97±3% of glucagon^+^ cells were found labeled with Arx (**D, F**), only 30±6% of glucagon^+^ cells appeared Arx^+^ in the Glu-ArxKO pancreata (**G, I**), suggesting an efficient deletion of *Arx* in approximately 70% of glucagon-producing cells. Interestingly, while Pax4 was not detected in α-cells (**E–F**) in controls, a small number of Arx^−^ glucagon^+^ cells were found to be Pax4^+^ (12±9% of glucagon cells) in Glu-ArxKO pancreata (**H–I**), indicating an ectopic expression of *Pax4* in glucagon^+^ cells upon *Arx* deficiency. (**J**) Using qPCR, a significant 74% reduction in the *Arx* transcript content was noted in Glu-ArxKO as compared to controls.
(TIF)

Figure S3 Validation of the IndGlu-ArxKO mouse line. Pancreata of 3 month-old controls (**A–C**) and 1mDox+ IndGlu-ArxKO (**D–F**) were subjected to quantitative analyses using the co-detection of Arx, Pax4 and glucagon. In the control pancreata, 98±2% of glucagon^+^ cells were found to be labeled with Arx (**A–C)**, whilst only 11±7% of glucagon^+^ cells appeared to be Arx^+^ in Dox+ IndGlu-ArxKO pancreata (**D–F**), suggesting an efficient deletion of *Arx* in approximately 90% of glucagon-expressing cells upon Dox treatment. Interestingly, though no expression of Pax4 was observed in glucagon^+^ cells in control pancreata (**B–C**), a small number of Arx^−^ glucagon^+^ cells were found to be Pax4^+^ (16±4% of glucagon^+^ cells) in Dox+ IndGlu-ArxKO animals (**E–F**), suggesting an ectopic expression of *Pax4* in such glucagon^+^ cells upon *Arx* deficiency.
(TIF)

Figure S4 Analysis of key β-cell and pan-endocrine markers following *Arx* inactivation in glucagon-expressing cells. Representative images of immunohistochemical analyses performed on islets of 7 month-old WT controls (**A–B, G–H, M–N, S–T**), 7 month-old Glu-ArxKO (**C–D, I–J, O–P, U–V**) and age-matched 5mDox+ IndGlu-ArxKO (**E–F, K–L, Q–R, W–X**) using the indicated antibody combinations. All insulin^+^ cells in all animals uniformly expressed the β-cell markers MafA (**A–F**), NeuroD1 (**G–L**) and HB9 (**M–R**), all endocrine cells being positive for the pan-endocrine marker Pax6 (**S–X**).
(TIF)

Figure S5 Quantification of endocrine cells in Glu-ArxKO and IndGlu-ArxKO pancreata. Quantitative comparison of the numbers of insulin- (**A**), glucagon- (**B**) and somatostatin- (**C**) expressing cells between 6 month-old Glu-ArxKO, 4mDox+ IndGlu-ArxKO and age-matched WT mice. A significant increase in the numbers of insulin- and somatostatin-expressing cells was observed in both transgenic lines compared to controls, while variations were noted in the number of glucagon-expressing cells. n = 3, ** p<0.01 using ANOVA.
(TIF)

Movie S1 OPT examinations. Pancreata from 5 month-old Glu-ArxKO animals (**Bottom**) and of age-/sex-matched WT controls [23] (**Top**) were stained with an anti-insulin antibody and visualized using Optical Projection Tomography to highlight the islet hypertrophy and increase in islet number upon *Arx* inactivation in glucagon-expressing cells.
(MOV)

Table S1 Assessment of the life expectancy, glycemic levels, islet size, and islet number in Glu-ArxKO animals. Glu-ArxKO mice were examined at the indicated ages. Life expectancy and basal glycemia (monitored weekly) were found within normal ranges, as compared to controls. Glu-ArxKO animals displayed a clear increase in islet size dependent on age, however this increase appeared to plateau at approximately 4 months of age. An increase in the islet number was also observed, suggestive of islet neogenesis, which peaked at an average of x1.98 compared to controls.
(DOCX)

Table S2 Assessment of the life expectancy, glycemic levels, islet size, and islet number in IndGlu-ArxKO animals. The IndGlu-ArxKO mice were treated with Dox at different ages and were examined after the indicated lengths of Dox treatment (values sorted based on Dox treatment duration). Life expectancy and basal glycemia (monitored weekly) were found within normal ranges, as compared to controls, in all conditions analyzed. Interestingly, a near doubling (1.8×) in islet size was observed after just 14 days of Dox with a steady increase in islet size dependent on length of Dox treatment until a plateau appeared to be reached after approximately 4 months of Dox treatment. No large variations were observed in islet number between the different durations of Dox treatment however, on average, a 1.9-fold increase was observed in Dox+ IndGlu-Arx animals compared to age-matched controls.
(DOCX)

Table S3 Quantification of glucagon^+^ cell-derived β-like cells. Using immunohistochemical analysis on Glu-ArxKO and Dox+ IndGlu-ArxKO pancreata, insulin^−^ and insulin^+^ β-gal-producing cells were manually counted and compared to controls. To determine the proportion of supplementary β-like cells expressing the β-gal tracer, the increase in β-like cell number was factored in using the following formula: (% of labeled β-cell×β-like cell increase)/(β-like cell increase - 1). Our data demonstrate a similar contribution (between 30 to 41%) of α-cells to the supplementary β-like cell count across models and indicated conditions.
(DOCX)
