# Supplementary figures and images for: The Inactivation of Arx in Pancreatic α-Cells Triggers Their Neogenesis and Conversion into Functional β-Like Cells
Source: PLoS Genet. 2013 Oct 31;9(10):e1003934. doi: 10.1371/journal.pgen.1003934 (PMC3814322; doi:10.1371/journal.pgen.1003934)

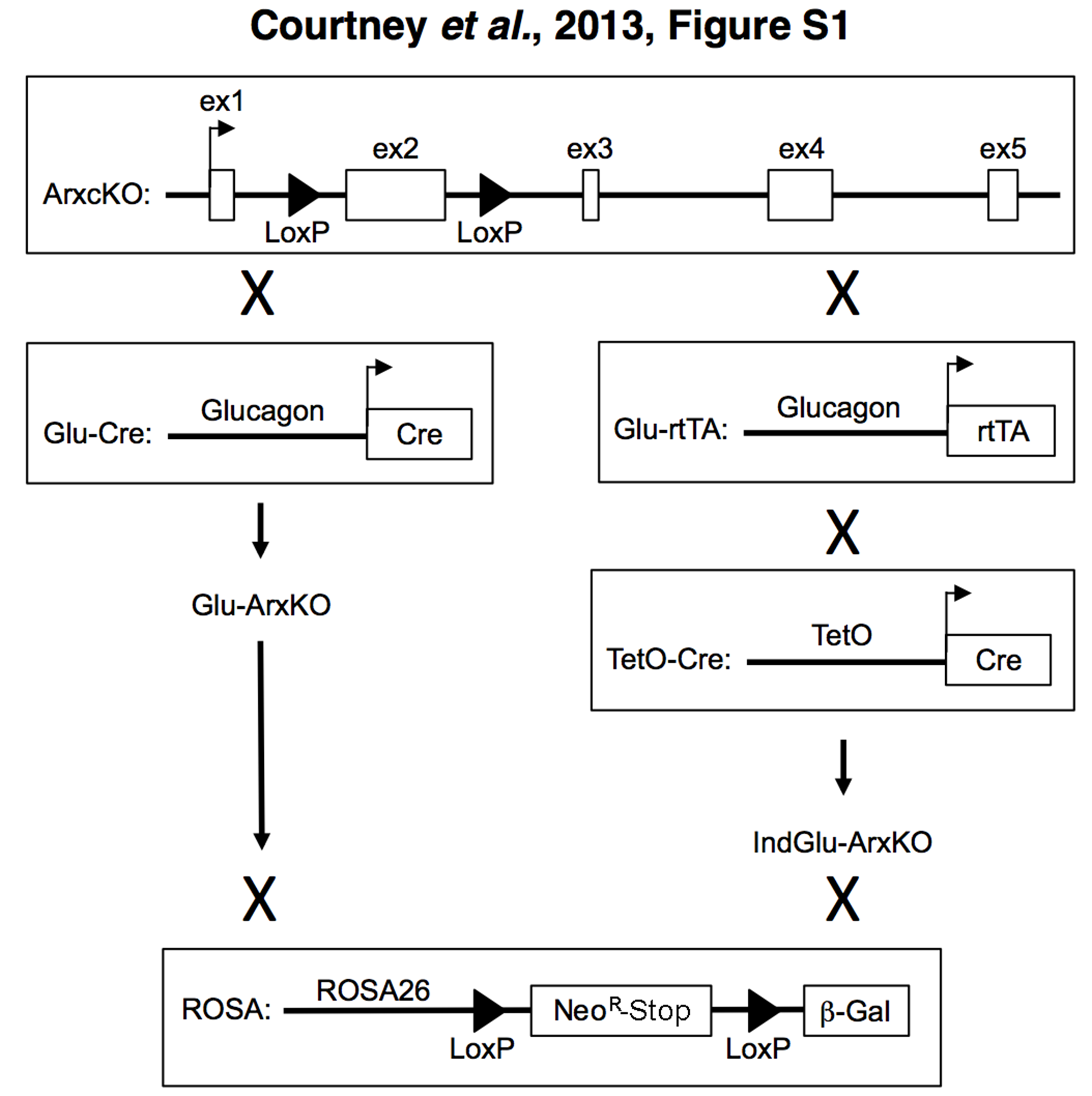

Supplement: Figure S1 — Generation of Glu-ArxKO and IndGlu-ArxKO animals. (Left) ArxcKO animals (in which the second exon of the Arx gene is flanked by LoxP sites) were crossed with the Glu-Cre mouse line (generated using a transgene composed of the glucagon promoter driving the expression of the phage P1 Cre recombinase). The resulting double transgenics (referred to as Glu-ArxKO) were further crossed with ROSA26-LoxP-Neomycin Resistance-STOP-LoxP-β-gal animals (“Rosa”, containing a transgene encompassing the ubiquitous ROSA26 promoter in front of the neomycin resistance gene with a STOP codon flanked by LoxP sites and followed by the β-galactosidase cDNA) for lineage tracing purposes. (Right) In a second mating scheme, ArxcKO animals were mated to Glu-rtTA mice (containing a transgene composed of the rat glucagon promoter upstream of the reverse tetracycline-dependent transactivator) and further mated with TetO-Cre animals (whose transgene includes the Tet operator upstream of Cre Recombinase cDNA). The resulting triple-transgenic mice, referred to as IndGlu-ArxKO, were subsequently mated with Rosa animals for lineage tracing purposes (IndGlu-ArxKO::Rosa). (TIF) [file pgen.1003934.s001.tif]

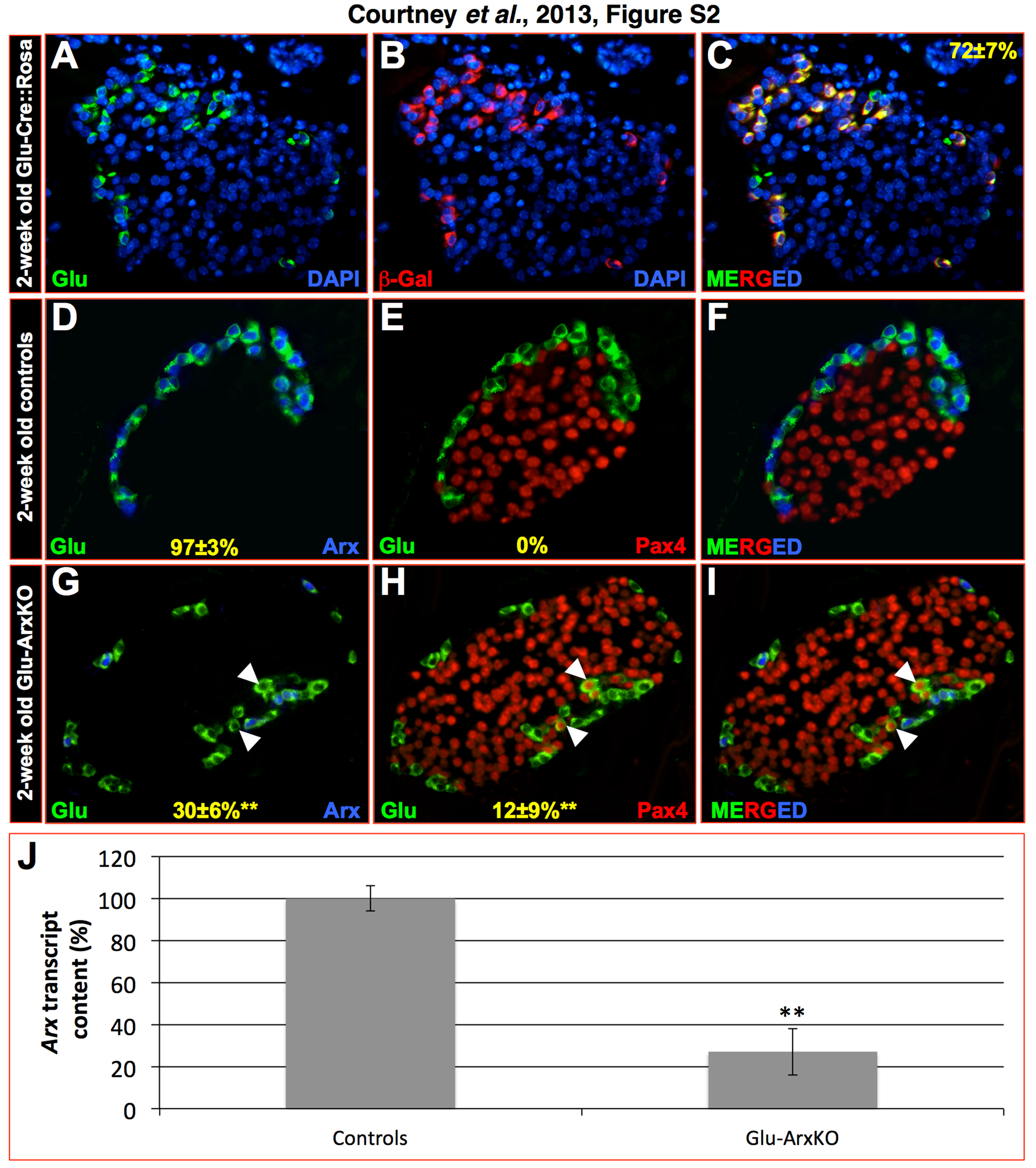

Supplement: Figure S2 — Validation of the Glu-ArxKO mouse line. (A–C) Aiming to further demonstrate the efficiency of the glucagon-mediated expression of the Cre recombinase, Glu-Cre animals were initially crossed to Rosa mice. Using immunohistochemistry with antibodies raised against glucagon or β-galactosidase, we showed a clear co-expression of the β-galactosidase with the glucagon hormone. In fact, quantitative analyses showed that 72% of glucagon-producing cells were β-galactosidase+, further demonstrating the relatively high efficiency of the Cre recombinase expression in this cell subtype. (D–I) Pancreata of 2 month-old controls (D–F) and of Glu-ArxKO (G–I) animals were subjected to quantitative analyses using a co-detection of Arx, Glucagon and Pax4. While in control pancreata, 97±3% of glucagon+ cells were found labeled with Arx (D, F), only 30±6% of glucagon+ cells appeared Arx+ in the Glu-ArxKO pancreata (G, I), suggesting an efficient deletion of Arx in approximately 70% of glucagon-producing cells. Interestingly, while Pax4 was not detected in α-cells (E–F) in controls, a small number of Arx− glucagon+ cells were found to be Pax4+ (12±9% of glucagon cells) in Glu-ArxKO pancreata (H–I), indicating an ectopic expression of Pax4 in glucagon+ cells upon Arx deficiency. (J) Using qPCR, a significant 74% reduction in the Arx transcript content was noted in Glu-ArxKO as compared to controls. (TIF) [file pgen.1003934.s002.tif]

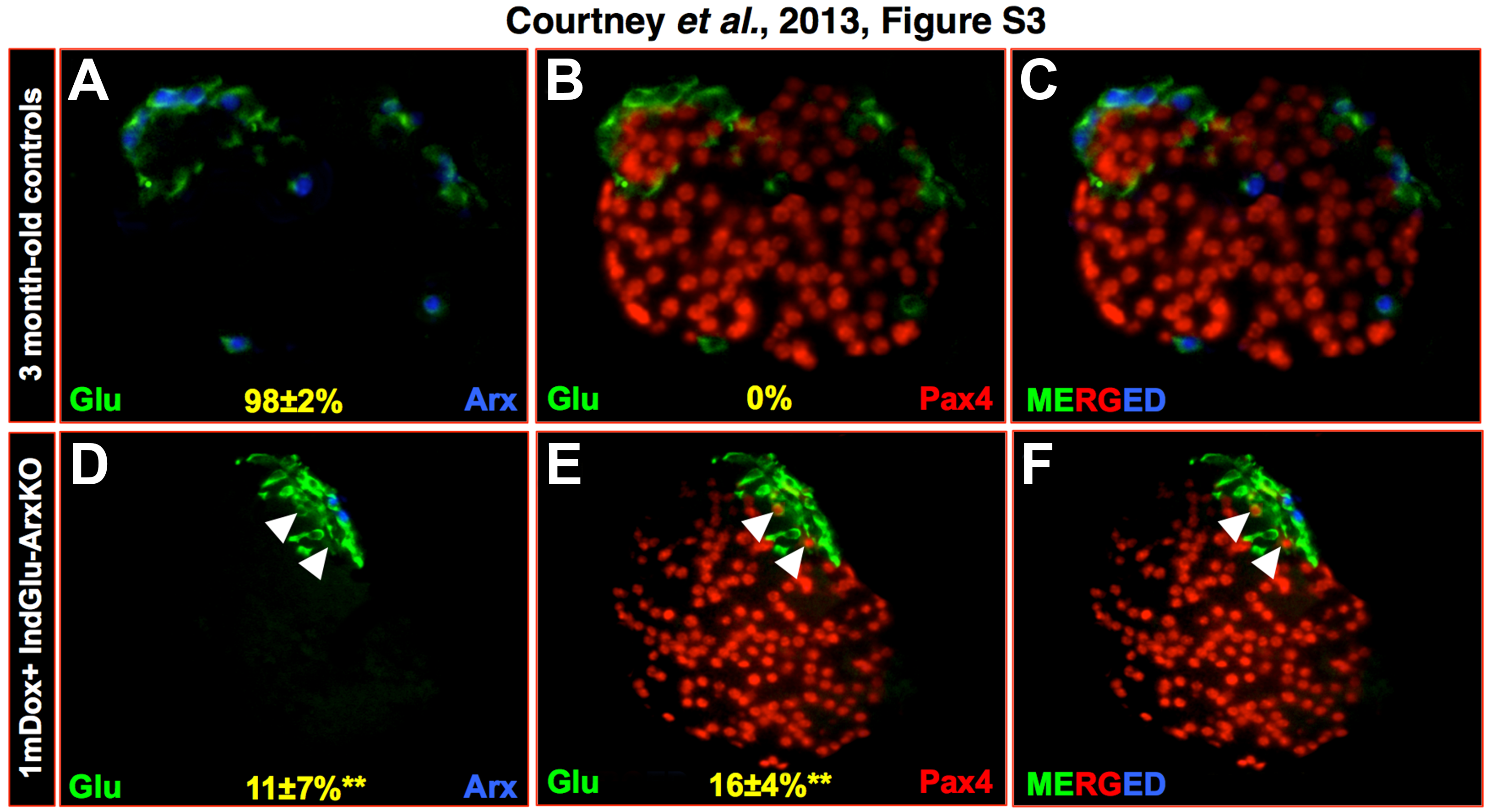

Supplement: Figure S3 — Validation of the IndGlu-ArxKO mouse line. Pancreata of 3 month-old controls (A–C) and 1mDox+ IndGlu-ArxKO (D–F) were subjected to quantitative analyses using the co-detection of Arx, Pax4 and glucagon. In the control pancreata, 98±2% of glucagon+ cells were found to be labeled with Arx (A–C), whilst only 11±7% of glucagon+ cells appeared to be Arx+ in Dox+ IndGlu-ArxKO pancreata (D–F), suggesting an efficient deletion of Arx in approximately 90% of glucagon-expressing cells upon Dox treatment. Interestingly, though no expression of Pax4 was observed in glucagon+ cells in control pancreata (B–C), a small number of Arx− glucagon+ cells were found to be Pax4+ (16±4% of glucagon+ cells) in Dox+ IndGlu-ArxKO animals (E–F), suggesting an ectopic expression of Pax4 in such glucagon+ cells upon Arx deficiency. (TIF) [file pgen.1003934.s003.tif]

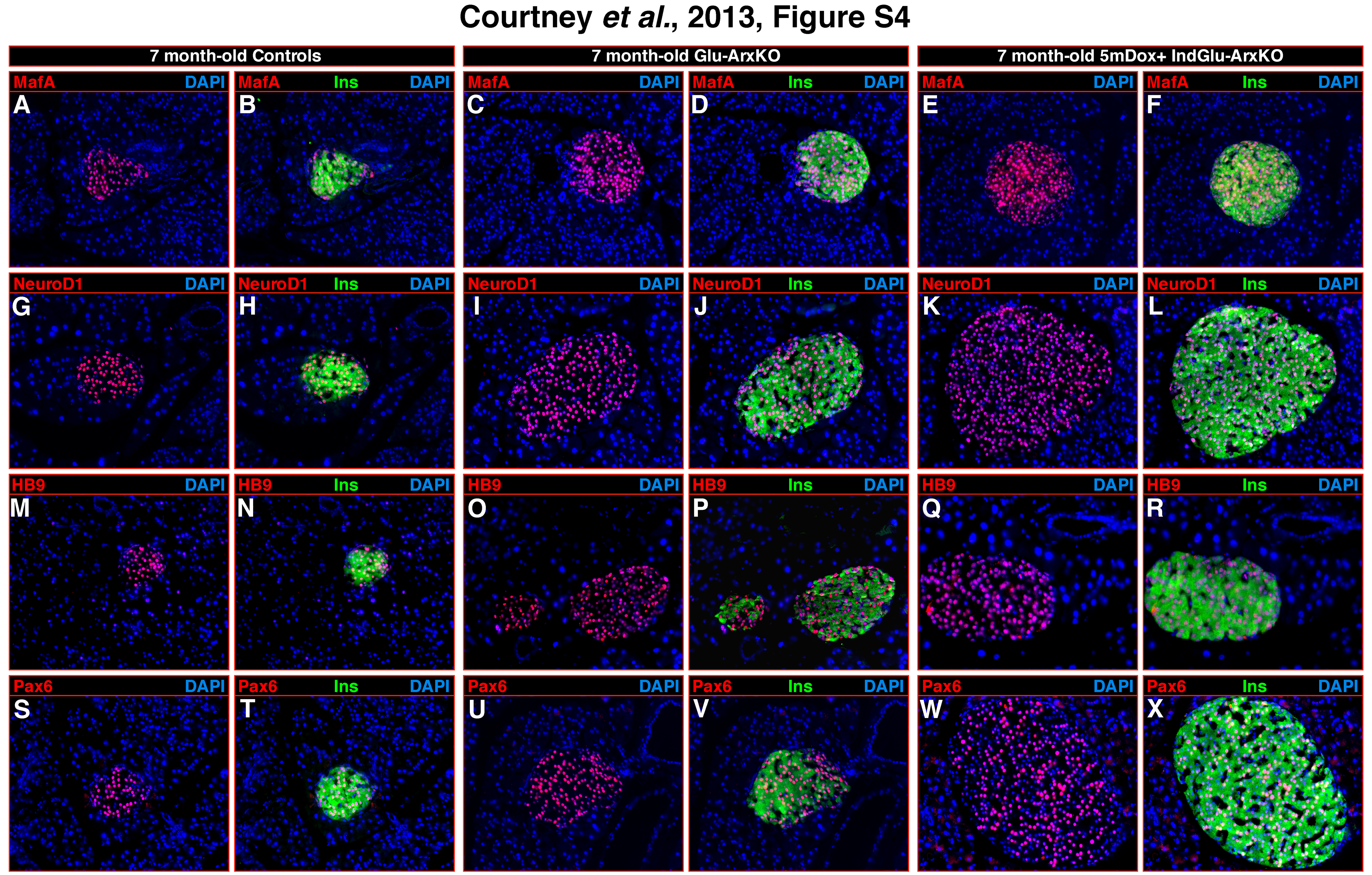

Supplement: Figure S4 — Analysis of key β-cell and pan-endocrine markers following Arx inactivation in glucagon-expressing cells. Representative images of immunohistochemical analyses performed on islets of 7 month-old WT controls (A–B, G–H, M–N, S–T), 7 month-old Glu-ArxKO (C–D, I–J, O–P, U–V) and age-matched 5mDox+ IndGlu-ArxKO (E–F, K–L, Q–R, W–X) using the indicated antibody combinations. All insulin+ cells in all animals uniformly expressed the β-cell markers MafA (A–F), NeuroD1 (G–L) and HB9 (M–R), all endocrine cells being positive for the pan-endocrine marker Pax6 (S–X). (TIF) [file pgen.1003934.s004.tif]

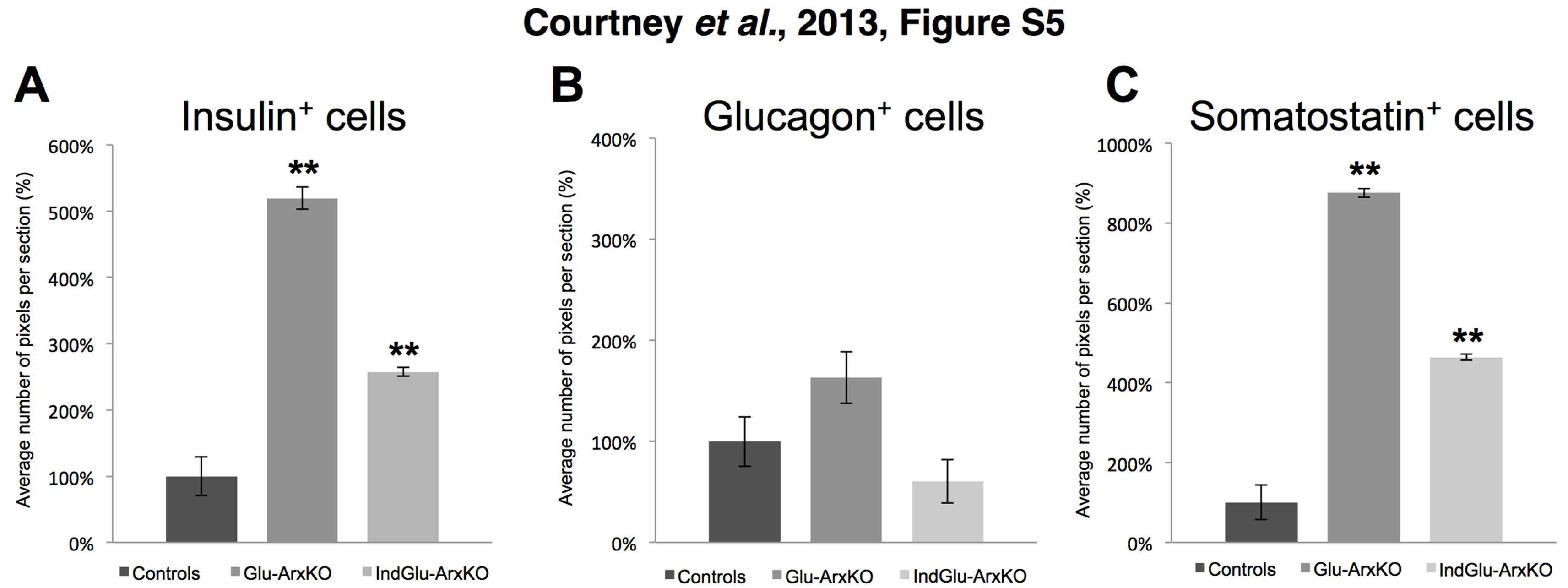

Supplement: Figure S5 — Quantification of endocrine cells in Glu-ArxKO and IndGlu-ArxKO pancreata. Quantitative comparison of the numbers of insulin- (A), glucagon- (B) and somatostatin- (C) expressing cells between 6 month-old Glu-ArxKO, 4mDox+ IndGlu-ArxKO and age-matched WT mice. A significant increase in the numbers of insulin- and somatostatin-expressing cells was observed in both transgenic lines compared to controls, while variations were noted in the number of glucagon-expressing cells. n = 3, ** p<0.01 using ANOVA. (TIF) [file pgen.1003934.s005.tif]
