# Supplementary material for: The Inactivation of Arx in Pancreatic α-Cells Triggers Their Neogenesis and Conversion into Functional β-Like Cells
Source: PLoS Genet. 2013 Oct 31;9(10):e1003934. doi: 10.1371/journal.pgen.1003934 (PMC3814322; doi:10.1371/journal.pgen.1003934)
Supplement: Table S1 — Assessment of the life expectancy, glycemic levels, islet size, and islet number in Glu-ArxKO animals. Glu-ArxKO mice were examined at the indicated ages. Life expectancy and basal glycemia (monitored weekly) were found within normal ranges, as compared to controls. Glu-ArxKO animals displayed a clear increase in islet size dependent on age, however this increase appeared to plateau at approximately 4 months of age. An increase in the islet number was also observed, suggestive of islet neogenesis, which peaked at an average of x1.98 compared to controls. (DOCX) [file pgen.1003934.s007.docx]

**Courtney *et al.*, 2013 - Table S1**

| **Glu-ArxKO animals** | | | | | |
| --- | --- | --- | --- | --- | --- |
| **Age of examination** | **Life expectancy** | **Basal glycaemia** | **Islet count**  **versus age-**  **/sex-matched controls** | **Islet size**  **versus age-**  **/sex-matched controls** |  |
| 2.4 months | Normal | 147 | x 1.4 | x 1.8 |  |
| 4 months | Normal | 121 | x 1.7 | x 3.3 |  |
| 6.1 months | Normal | 152 | x 2.6 | x 3.4 |  |
| 7.2 months | Normal | 141 | x 2.1 | x 3.3 |  |
| 11.4 months | Normal | 140 | x 2.1 | x 3 |  |
