# Supplementary material for: The Inactivation of Arx in Pancreatic α-Cells Triggers Their Neogenesis and Conversion into Functional β-Like Cells
Source: PLoS Genet. 2013 Oct 31;9(10):e1003934. doi: 10.1371/journal.pgen.1003934 (PMC3814322; doi:10.1371/journal.pgen.1003934)
Supplement: Table S2 — Assessment of the life expectancy, glycemic levels, islet size, and islet number in IndGlu-ArxKO animals. The IndGlu-ArxKO mice were treated with Dox at different ages and were examined after the indicated lengths of Dox treatment (values sorted based on Dox treatment duration). Life expectancy and basal glycemia (monitored weekly) were found within normal ranges, as compared to controls, in all conditions analyzed. Interestingly, a near doubling (1.8×) in islet size was observed after just 14 days of Dox with a steady increase in islet size dependent on length of Dox treatment until a plateau appeared to be reached after approximately 4 months of Dox treatment. No large variations were observed in islet number between the different durations of Dox treatment however, on average, a 1.9-fold increase was observed in Dox+ IndGlu-Arx animals compared to age-matched controls. (DOCX) [file pgen.1003934.s008.docx]

**Courtney *et al.*, 2013 - Table S2**

| **Dox+ IndGlu-ArxKO animals** | | | | | | |
| --- | --- | --- | --- | --- | --- | --- |
| **Dox Treatment Age** | **Dox Treatment Duration** | **Life expectancy** | **Basal glycaemia** | **Islet count**  **versus age-**  **/sex-matched controls** | **Islet size**  **versus age-**  **/sex-matched controls** |  |
| 2.2 months | 14 days | Normal | 135 | x 1.5 | x 1.8 |  |
| 2.3 months | 1 month | Normal | 153 | x 1.2 | x 1.6 |  |
| 1.8 months | 2.6 months | Normal | 151 | x 2.3 | x 2.4 |  |
| 2 months | 3.8 months | Normal | 164 | x 2.6 | x 3.4 |  |
| 5.7 months | 4.1 months | Normal | 136 | x 1.6 | x 2.1 |  |
| 2.5 months | 4.8 months | Normal | 134 | x 2.4 | x 2.8 |  |
| 9 months | 5.5 months | Normal | 119 | x 1.7 | x 3.1 |  |
| 2 months | 5.8 months | Normal | 163 | x 2.1 | x 2.7 |  |
| 2.3 months | 10.3 months | Normal | 139 | x 2.4 | x 3.3 |  |
