# Supplementary material for: The Inactivation of Arx in Pancreatic α-Cells Triggers Their Neogenesis and Conversion into Functional β-Like Cells
Source: PLoS Genet. 2013 Oct 31;9(10):e1003934. doi: 10.1371/journal.pgen.1003934 (PMC3814322; doi:10.1371/journal.pgen.1003934)
Supplement: Table S3 — Quantification of glucagon+ cell-derived β-like cells. Using immunohistochemical analysis on Glu-ArxKO and Dox+ IndGlu-ArxKO pancreata, insulin− and insulin+ β-gal-producing cells were manually counted and compared to controls. To determine the proportion of supplementary β-like cells expressing the β-gal tracer, the increase in β-like cell number was factored in using the following formula: (% of labeled β-cell×β-like cell increase)/(β-like cell increase - 1). Our data demonstrate a similar contribution (between 30 to 41%) of α-cells to the supplementary β-like cell count across models and indicated conditions. (DOCX) [file pgen.1003934.s009.docx]

**Courtney *et al.*, 2013 - Table S3**

| **(Treatment) Genotype** | **Labeled β-cells (%)** | | **β-like cell increase**  **versus controls** | **Labeled Supplementary**  **β-like cells (%)** | | |
| --- | --- | --- | --- | --- | --- | --- |
| 2.4m Glu-ArxKO | 14.37±2.12 | x1.8 | | | 32.33±4.77 |  |
| 3.4m Glu-ArxKO | 28.35±3.41 | x3.3 | | | 40.68±4.90 |  |
| 8.2m Glu-ArxKO | 26.30±1.98 | x3.3 | | | 37.73±2.85 |  |
| 2.6mDox+ IndGlu-ArxKO | 17.91±1.77 | x2.4 | | | 30.70±3.03 |  |
| 4.1mDox+ IndGlu-ArxKO | 20.33±2.44 | x2.1 | | | 38.81±4.67 |  |
